# Toward Bright Polymetallic Biopolymer-Protected Luminescent Nanoclusters

**DOI:** 10.3390/molecules31183238

**Published:** 2026-09-13

**Authors:** Andrey A. Buglak, Varvara G. Kubenko, Nikolay V. Shekhovtsov, Tomash S. Sych, Alexei I. Kononov

**Affiliations:** Faculty of Physics, Saint-Petersburg State University, Universitetskaya Embankment 7-9, 199034 St. Petersburg, Russia; andreybuglak@gmail.com (A.A.B.); st063382@student.spbu.ru (V.G.K.); n.shekhovtsov@spbu.ru (N.V.S.); t.sych@spbu.ru (T.S.S.)

**Keywords:** nanoclusters, bimetallic nanoclusters, doping, luminescence enhancement, quantum yield, excited state dynamics, biosensor

## Abstract

Polymetallic nanoclusters (NCs) offer a powerful route to brighter and more tunable luminescent materials, while biopolymer ligands provide additional advantages for sensing and bioimaging. However, the mechanisms responsible for luminescence enhancement remain poorly understood, particularly for DNA- and protein-protected NCs. Here, we critically review the relationship between metal composition, cluster structure, ligand environment, and excited-state dynamics in luminescent polymetallic NCs. Rather than treating alloying as a single “synergistic” effect, we distinguish three recurrent contributions to enhanced emission: suppression of nonradiative relaxation through structural or ligand-shell rigidification, modification of the electronic structure and radiative transitions, and enhanced intersystem crossing in phosphorescent systems. Particular attention is given to cases in which changes in quantum yield can be interpreted together with excited-state lifetimes and other spectroscopic data, allowing experimentally supported mechanisms to be distinguished from plausible but unverified explanations. We further evaluate the performance of biopolymer-protected polymetallic NCs in sensing and related applications and identify key limitations, including the scarcity of atomic structures and systematic excited-state measurements. This analysis provides a framework for connecting metal composition and molecular structure to luminescence and highlights strategies for the rational development of brighter, more stable, and biocompatible NCs.

## 1. Introduction

Metal nanoclusters (NCs), typically composed of only a few to several tens of metal atoms, occupy an intermediate regime between molecular complexes and larger nanoparticles. Their ultrasmall dimensions and quantum-confined electronic structures give rise to discrete energy levels and molecular-like optical properties, including size- and composition-dependent luminescence. In contrast to conventional fluorophores, NCs can combine high photoluminescence brightness with structural tunability and pronounced sensitivity to their chemical environment, making them attractive for applications in sensing, bioimaging, and other optical technologies [1,2]. Here, brightness is particularly important because it is determined by both the efficiency of light absorption and the photoluminescence quantum yield (QY). For bioimaging and sensing, however, high brightness alone is insufficient: photostability is equally important for maintaining a reliable optical signal under prolonged excitation. Although metal NCs can also exhibit remarkable catalytic properties, this review focuses specifically on their luminescent behavior.

**Figure 1:**
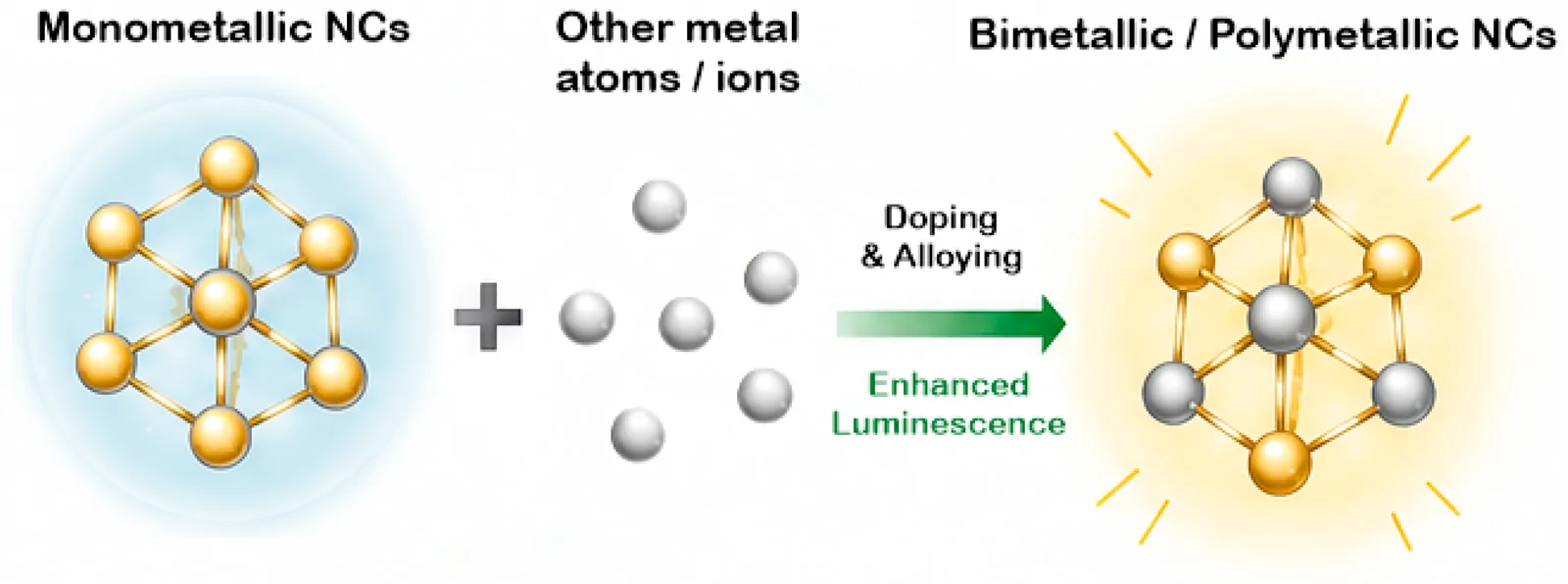
Conceptual scheme illustrating the formation of a bimetallic NC with enhanced luminescence from a monometallic NC by incorporation of atoms or ions of another metal.

One promising strategy for increasing the brightness of metal NCs is the incorporation of two or more metal components. Such bimetallic and polymetallic NCs, often described as alloyed or doped clusters depending on their structure and composition, provide additional degrees of freedom for tuning the electronic structure, geometry, and excited-state dynamics. Over the past years, numerous polymetallic NCs have been synthesized, and many exhibit pronounced changes in their optical properties, including enhanced luminescence compared with their monometallic counterparts (Figure 1). These systems include bimetallic Au-Ag [3,4,5,6,7,8,9,10,11], Au-Cu [12,13], and Ag-Pt/Pd [14,15] clusters, as well as trimetallic Au-Ag-M systems [16,17,18] and tetrametallic [19] NCs. Several synthetic strategies have been developed for preparing such clusters. In direct co-reduction, different metal precursors are combined with stabilizing ligands and subsequently reduced to form polymetallic NCs. This approach has enabled the preparation of a wide range of bimetallic and higher-order polymetallic clusters, including Au/Ag systems originating from the pioneering work of Teo and Keating [20]. Depending on the metal combination and reaction conditions, co-reduction can produce clusters with substantially different structural and optical properties from their monometallic analogues. For example, ligand engineering was used to enhance the luminescence of Au_2_Cu_6_ NCs [21], while Au doping of Ag NCs produced a 26-fold increase in luminescence QY [6]. Other studies have demonstrated luminescence-related effects in Au-Ag alloy NCs through ligand-controlled electronic and structural changes [8,22] and aggregation-induced emission [23,24]. Other approaches include galvanic and anti-galvanic reduction, metal exchange, ligand exchange, and reactions between preformed NCs. A comprehensive overview of synthetic strategies for polymetallic NCs can be found elsewhere [25].

Importantly, alloying can produce a synergistic enhancement of luminescence that cannot be readily predicted from the properties of the individual metals. Several striking examples illustrate the potential of this approach. The QY of the Au_25_ cluster [Au_25_(PPh_3_)_10_(SR)_5_Cl_2_]^2+^ was enhanced by approximately 200-fold upon substitution of 13 Au atoms with Ag, with the resulting cluster exhibiting a luminescence QY of up to 40% [10]. Conversely, Ag_29_ doped with Au showed a 26-fold enhancement in emission [6]. A rod-shaped trimetallic Pt/Au/Ag cluster exhibited a luminescence QY of 14.7% [26], while Pt_2_Au_10_Ag_13_ displayed a QY of approximately 15%, about 150 times higher than that of its bimetallic Pt_1_Ag_24_ counterpart [17]. Particularly remarkable results were reported for Au_16_Cu_6_, for which a phosphorescence QY approaching unity was achieved together with an approximately 300-fold enhancement of intersystem crossing [27]. These examples demonstrate that incorporation of additional metal components can profoundly alter the photophysics of NCs and can provide a powerful route toward substantially brighter emitters.

**Figure 2:**
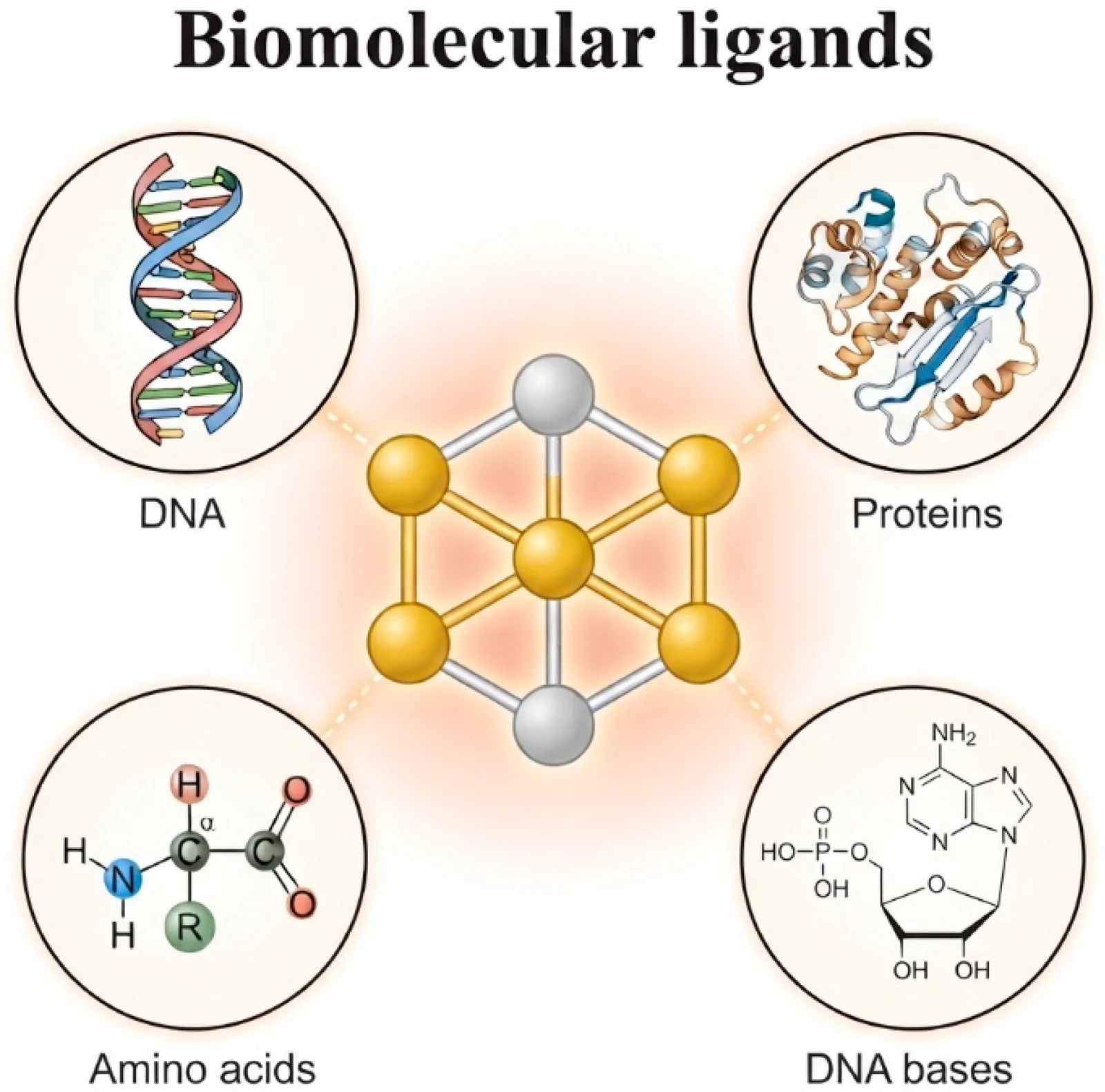
Bimetallic nanoclusters stabilized by biomolecules: enhanced luminescence and biosensing potential.

The ligand environment is another key factor governing the structure and photophysical properties of metal NCs. Conventional solution-phase syntheses commonly employ small organic ligands, polymers, or surfactants, including thiols, phosphines, polyvinylpyrrolidone (PVP), and polyethylene glycol (PEG). Although such ligands can provide effective stabilization and control over cluster growth, biomolecular ligands offer additional advantages for applications in bioimaging and biosensing. DNA, proteins, peptides, and amino acids can provide biocompatibility, low toxicity, and specific molecular recognition, while their diverse binding sites and conformational properties can influence metal-cluster nucleation, structure, and electronic states (Figure 2). In addition, selective host–guest and biomolecular recognition interactions can be exploited to construct NC-based sensors with enhanced molecular specificity.

Thiol-protected NCs, particularly those stabilized by glutathione (GSH), provide an important bridge between conventional ligand-protected clusters and biomolecularly stabilized systems. Thiol-containing ligands are capable of coordinating metal atoms and can therefore serve as a model for the interactions of metal clusters with sulfur-containing peptides and proteins. Starting from monometallic Au [28,29,30], Ag [31,32], and Cu [33] NCs, bimetallic Au-Ag [3,4,9,11], Au-Cu [13], and Ag-Pd/Pt [14] nanoclusters have subsequently been synthesized, followed by the development of trimetallic Au-Ag-M systems [18]. Because the atomic structures of many thiolate-protected NCs have been determined, these systems provide valuable model platforms for establishing relationships between metal composition, cluster structure, ligand interactions, and luminescence.

Despite the rapid growth of the literature, a critical analysis of why polymetallic NCs become more luminescent is still lacking. Previous reviews have largely focused on the synthesis, structural diversity, general optical properties, or applications of atomically precise and biopolymer-stabilized NCs. In contrast, the present review focuses specifically on the mechanistic origin of luminescence enhancement and on the extent to which proposed mechanisms are supported by experimental evidence. We distinguish effects associated with structural rigidification and suppression of nonradiative relaxation from changes in electronic structure and radiative transitions, as well as from enhanced intersystem crossing in phosphorescent systems. Particular emphasis is placed on comparisons between monometallic and polymetallic analogues and on studies combining quantum yields with excited-state lifetimes, ultrafast spectroscopy, structural characterization, and theoretical calculations. For biopolymer-protected NCs, where atomic structures are often unavailable, we explicitly identify the limits of mechanistic interpretation and distinguish established observations from hypotheses. This framework allows the current state of the field to be evaluated not only in terms of reported luminescence enhancement, but also in terms of how convincingly its molecular origin has been established.

In this review, we first consider polymetallic NCs stabilized by thiolate ligands, with emphasis on systems exhibiting particularly strong luminescence enhancement relative to their monometallic analogues. These structurally well-characterized clusters provide useful models for understanding how metal composition and ligand–cluster interactions control excited-state dynamics. We then turn to polymetallic NCs stabilized by biopolymers, focusing primarily on DNA- and protein-templated systems. We compare their luminescence properties with those of corresponding monometallic NCs and discuss the structural and photophysical factors responsible for emission enhancement. Because extinction coefficients for NCs are rarely reported, we focus primarily on QYs (or luminescence intensities when QYs are unavailable), rather than attempting to compare brightness. Applications of biopolymer-protected polymetallic NCs in sensing, bioimaging, and catalysis are also considered. Finally, we identify outstanding challenges in establishing clear structure–composition–photophysics relationships and discuss prospects for the rational design of brighter, more photostable, and biocompatible polymetallic nanoclusters.

## 2. NCs Stabilized by Thiolates

Some of the main potential applications of NCs are bioimaging and sensing. In this respect, the luminescent abilities of NCs come to the fore. The main efforts of researchers are aimed at synthesizing clusters with a high luminescence QY and identifying the factors affecting their luminescent properties. At the same time, even for monometallic NCs, the nature of luminescence often defies unambiguous interpretation. The luminescence QYs of most monometallic NCs are usually quite low. The synthesis of polymetallic NCs is a promising way to remedy this drawback.

As representative proteins, or more precisely peptides, GSH is probably one of the most frequently used thiol-containing biological templates for the synthesis of metal NCs. GSH-protected NCs are of particular interest since they are water-soluble, non-toxic, and can be used in biomedical applications. Thiol(SG)-templated NCs can emit from visible to near-infrared (NIR) spectral range with low QY. Blue-emitting Cu-SG NCs exhibit a QY of ca. 9% [33]. Blue-emitting Ag_12_(GSH)_10_ exhibits a QY of 15% [34]. Blue and Yellow AgGSH NCs reported in [32] have QYs of 60% and 68%, respectively. Red Ag-SG NCs are reported to have a QY of ca. 4% [35] and 2% [36]. Luminescent red Au−SG NCs with a QY of ∼15% were synthesized [37]. Red NIR-emitting Au-SG NCs were synthesized with a QY of ca. 10% [38]. Red Au_22_(SG)_18_ possesses a QY of ca. 8% [39]. The QY for similar gold NCs such as Au_18_(SG)_14_ (QY of 0.37%), Au_15_(SG)_13_ (QY of 0.025%), and Au_25_(SG)_18_ (QY of 0.12%) [40] and others is typically low.

**Figure 3:**
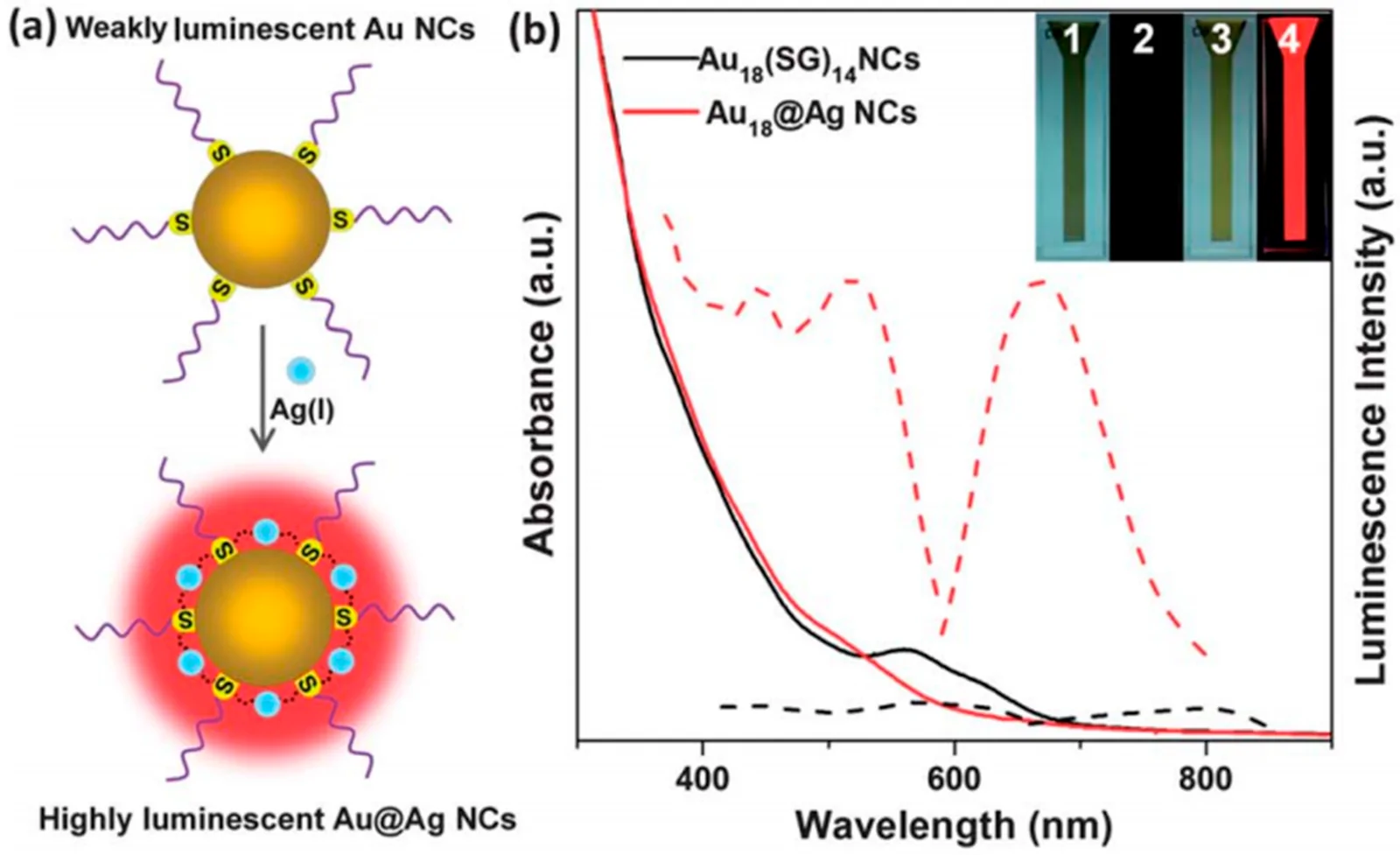
(**a**) Schematic illustration of the rigidifying ligand shell in Au_18_(SG)_14_ NCs by Ag(I) ions, which enhances luminescence. (**b**) Absorption (solid lines) and luminescence (dashed lines) spectra of the Au_18_(SG)_14_ (black lines) and Au@Ag (red lines) NCs; inset: digital photos of the Au_18_(SG)_14_ (1 and 2) and Au@Ag NCs (3 and 4) under visible (1, 3) and UV (2, 4) light. (Adopted under CC-BY 4.0 license http://creativecommons.org/licenses/by/4.0/ from ref. [40]).

Doping NCs with other noble metal atoms allows for an increase in the luminescence intensity and a shift in the emission wavelength. Xie et al. [40] studied the above-mentioned class of Au NCs–Au_15_, Au_18_, and Au_25_ based on GSH and doped them with silver ions. In doing so, silver does not substitute atoms in the metallic Au core but coordinates with sulfur in the S–Au–S staple motifs on the surface, forming Au@Ag structures (Figure 3). The QY enhancement factor was determined to be 84 for Au_15_@Ag, 18 for Au_18_@Ag, and 27 for Au_25_@Ag compared to their parent Au NCs [40]. The effect was explained by the so-called aggregation-induced emission (AIE). However, in absolute values, QYs remain quite low. For comparison, Au–thiolate NCs have been synthesized with a QY of ∼15% [37]. Rigidifying the ligand shell around the metal core is one of the strategies used to increase QY. For example, the QY of Au_22_(SG)_18_ increases up to >60% in toluene for a cluster rigidified with tetraoctylammonium [41]. A water-soluble analog of the Au_22_(SG)_18_ cluster, whose surface was modified with benzyl chloroformate and pyrene, had a QY of 30% [42]. In a bimetallic Au_2_Cu_6_ complex [43], Au^0^-induced aggregation of the CuSR complexes enhances luminescence up to 11.7%.

A series of studies on other thiolate-protected BNCs have been performed in recent decades. In this review, we concentrate on those whose luminescence was significantly enhanced upon alloying. It should be noted that practically all of them contain phosphines in stabilizing ligands.

**Figure 4:**
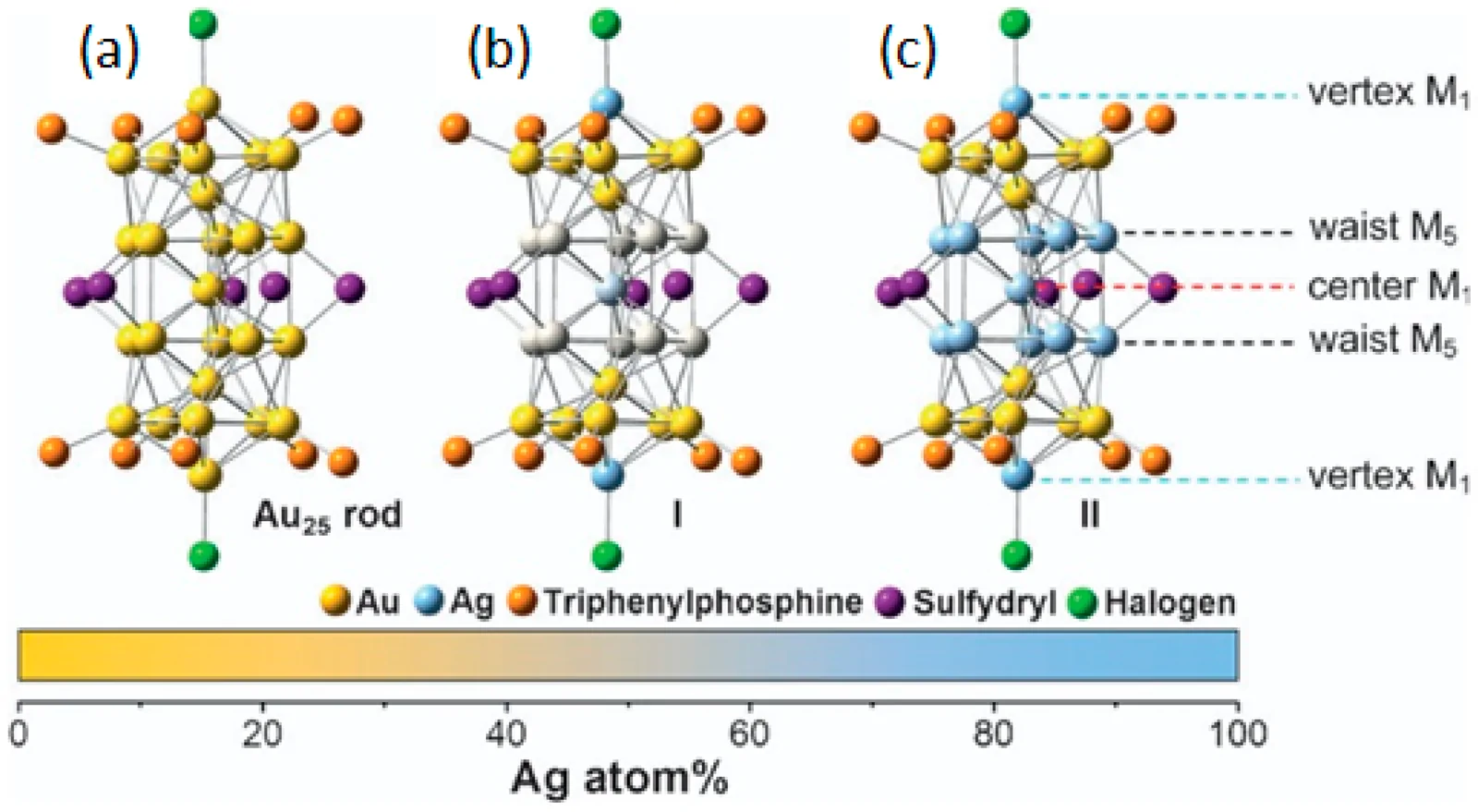
X-ray structures of the Au_25_ rod (**a**), Ag-doped clusters I (**b**) and II (**c**) (M = metal atom). (Reproduced with permission from [10]. Copyright 2014 Wiley-VCH GmbH).

For example, 25-atomic biicosahedral gold cluster [Au_25_(PPh_3_)_10_(SC_2_H_4_Ph)_5_Cl_2_]^2+^ exhibits a dramatic enhancement of its luminescence upon doping with silver atoms (Table 1). Ag_13_Au_12_ NCs demonstrated a remarkably high QY of 40% (λ_em_ ≈ 680 nm), which sharply contrasted with the typically weak fluorescence of similar Ag_x_Au_25−x_ NCs, but with an x of 1–12 (QY = 0.2%, λ_em._ ≈ 720 nm). This can be compared to a QY of ca. 0.1% of the monometallic Au_25_ NC (λ_em._ = 827 nm). The optical properties of the Au_25_ template revealed a pronounced sensitivity to the number of silver atoms integrated into the NC. Notably, the distinct Ag_13_Au_12_ composition in sample II (Figure 4) exhibited a significantly enhanced QY and blue-shifted luminescence. This incorporation of 13 silver atoms induced substantial alterations to the electronic structure of the Ag_13_Au_12_ NC. X-ray analysis of Ag-doped rod clusters showed that Ag atoms preferentially replace the two vertex Au atoms first, then the ten Au atoms in the waist region, and subsequently the central Au atom (Figure 4). Complete substitution of all 13 Au atoms resulted in strong perturbations to the electronic structure, which was evident in shifts in the electronic absorption spectra and increased luminescence intensity. In fact, silver substitution of Au atoms in NC results in the HOMO-LUMO transition involving only Ag 5s rather than mixed Au 6s and Ag 5s [10].

**Figure 5:**
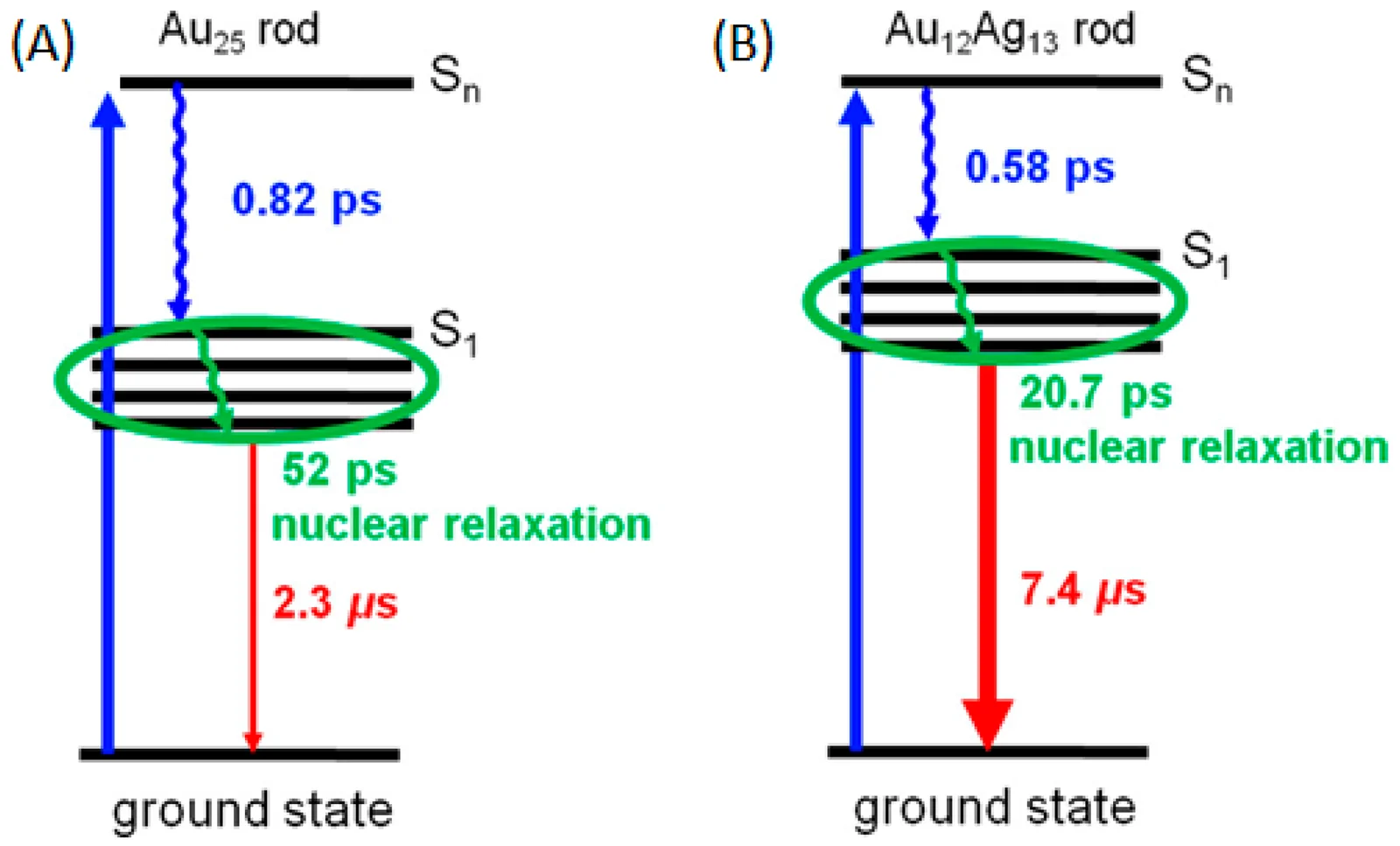
Excited state dynamics for the Au_25_ cluster (**A**) and the Au_12_Ag_13_ cluster (**B**). (Reproduced with permission from [44]. Copyright 2015 American Chemical Society).

Zhou et al. [44] studied the excited-state (ES) dynamics in the rod-shaped cluster [Au_25_(PPh_3_)_10_(SC_2_H_4_Ph)_5_Cl_2_]^2+^ and its highly luminescent Ag-alloyed counterpart Au_12_Ag_13_. Femtosecond transient absorption shows that in Au_12_Ag_13_, the relaxation proceeds via an ultrafast internal conversion (~0.58 ps), followed by nuclear (structural) relaxation (~20.7 ps), and then by a slow microsecond decay (~7.4 μs) back to the ground state. For the undoped Au_25_, the analogous nuclear step is substantially slower (~52 ps), indicating a “softer” structure and more efficient nonradiative channels. The authors calculated rate constants k_r_ and k_nr_ for both clusters. It appeared that for the Au_25_ rod k_r_ is 0.42 ms^−1^ and k_nr_ is 0.42 μs^−1^, where the nonradiative relaxation channel was dominated. While for the Au_12_Ag_13_ rod, k_r_ = 54 ms^−1^ and k_nr_ = 81 ms^−1^, which explained the increase in QY. The proposed photoprocesses in the cluster are shown in Figure 5.

**Figure 6:**
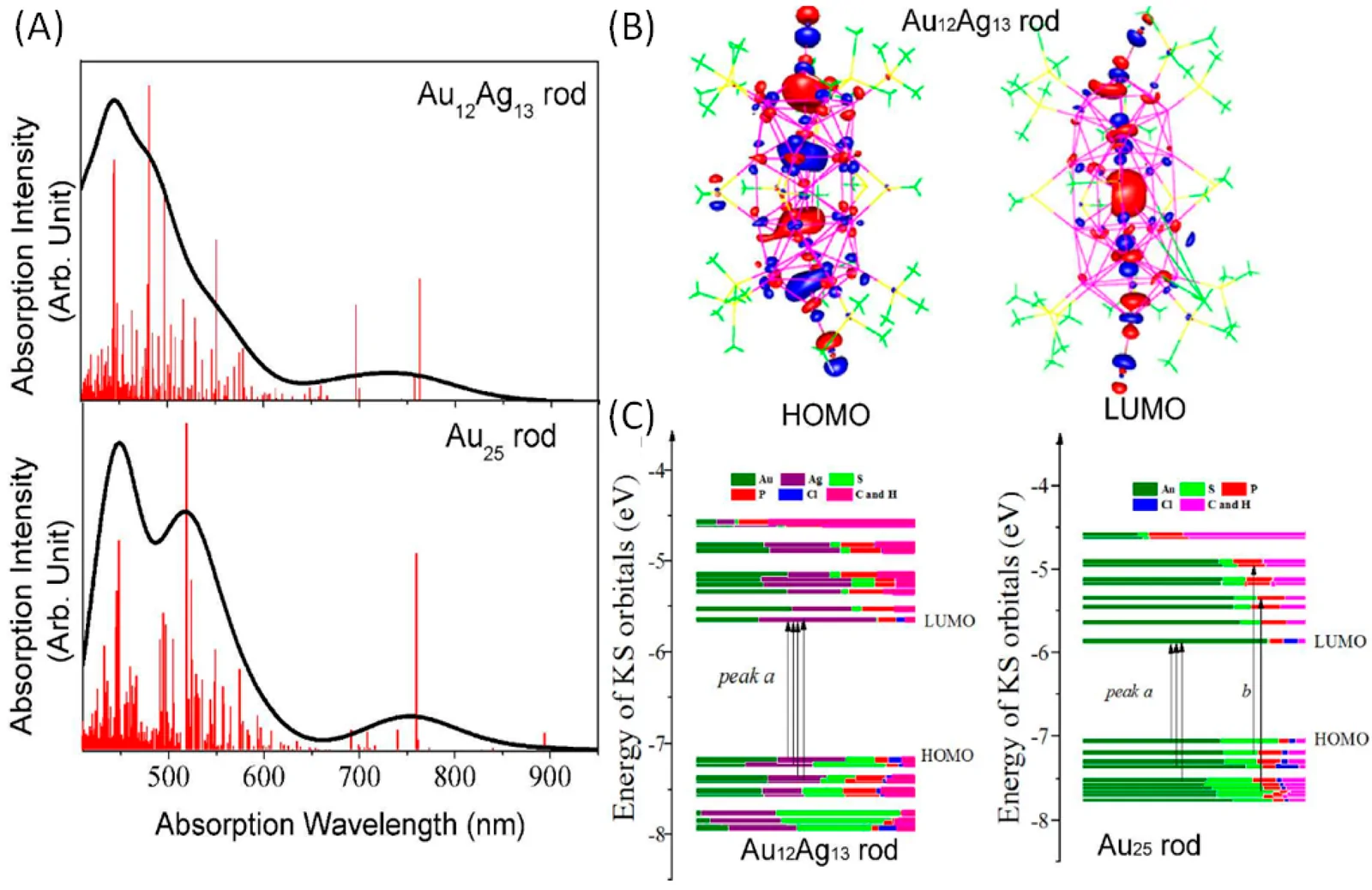
(**A**) Calculated absorption spectra of the Au_12_Ag_13_ rod and the Au_25_ rod; (**B**) electron density maps of molecular orbitals of the Au_12_Ag_13_ rod; (**C**) analysis of the orbital populations of the Au_12_Ag_13_ rod and the Au_25_ rod. (Reproduced with permission from [44]. Copyright 2015 American Chemical Society).

TD-DFT calculations demonstrate (Figure 6) that the central Ag atom stabilizes the LUMO, increases the HOMO–LUMO gap and thus blue-shifts the spectra, while simultaneously making the framework more rigid and suppressing nonradiative relaxation—this is directly linked to the increase in QY.

The work by Wu et al. [45] extends the understanding of the same rod-shaped Au_25_ and Au_12_Ag_13_ clusters, shifting the emphasis from purely electronic to mechanical properties and coherent vibrations. Using cryogenic UV–Vis spectroscopy, the authors show that the electron–phonon (electron–vibron) coupling in Au–Ag alloys has nearly the same average vibrational energy as in the pure Au_25_ rod, whereas the spherical Au_25_(SR)_18_ exhibits a higher vibrational energy, which is attributed to stronger core–shell interactions. Femtosecond and nanosecond transient absorption reveals pronounced coherent oscillations in both clusters with frequencies of ~0.9 THz (Au_25_) and ~0.65 THz (Au_12_Ag_13_), while during the oscillations the amplitude of the GSB/ESA signals changes, but their energy position does not. This allows the authors to interpret the observed behavior as an acoustic quadrupole-like extension mode along the long axis, implementing an amplitude (non-Condon) modulation of the transition rather than a modulation of the energy gap. An important conclusion by Wu et al. is that silver alloying, contrary to simple elastic models (where a lighter metal should accelerate the vibrations), leads to some slowing down and longer coherence. They attribute this to changes in the rod length and structure of the ES of the alloy relative to the pure Au cluster, as well as to the influence of the ligand shell, and show that coherent vibrational dynamics can be used to probe the mechanical properties of alloyed clusters.

In contrast to the above-mentioned Au_25_ cluster, another BNC, Au_25−n_Ag_n_(SR)_18_, where R = C_12_H_25_, with an icosahedral M_13_ inner core does not exhibit significant enhancement of luminescence. However, a gradual increment in the number of doping Ag atoms allowed to progressively blueshift the emission wavelength. This alteration in luminescence properties was attributed to some changes in the electronic structure and/or the surface state, which arose from the integration of silver atoms into the M_13_ core [46].

Doping of Ag NCs with Au, Pd or Pt atoms can also modify their luminescent properties. Thus, a specific number of Au atoms have been incorporated into the Ag_29_(BDT)_12_(TPP)_4_ cluster, achieving a remarkable enhancement in fluorescence QY by approximately 26 times, while preserving the geometrical integrity of the Ag_29_ NC. This controlled doping not only improved the luminescence intensity but also significantly enhanced the stability of the Ag NC [6]. The ES dynamics of the Ag_29_ NC exhibited a ~300-ns decay component referred to as a ligand-to-metal charge transfer (LMCT). In the AuAg NC, the dynamics showed the presence of two components with lifetimes of ca. 100 and 1000 ns. The authors attributed the changes in the ES dynamics to a change in the degree of the CT in the S1 state. It is worth noting that the average luminescence lifetime increased approximately twofold, while the QY exhibited enhancement by two orders more. This fact strongly suggests that the luminescent enhancement in the AuAg NCs involves a substantial increase in the radiative rate, rather than being explained solely by prolonged excited-state lifetimes or suppression of nonradiative relaxation. Consistent with this interpretation, quantum-chemical calculations predicted a substantial increase in the transition dipole moment upon alloying, which was attributed to relativistic effects [47].

**Figure 7:**
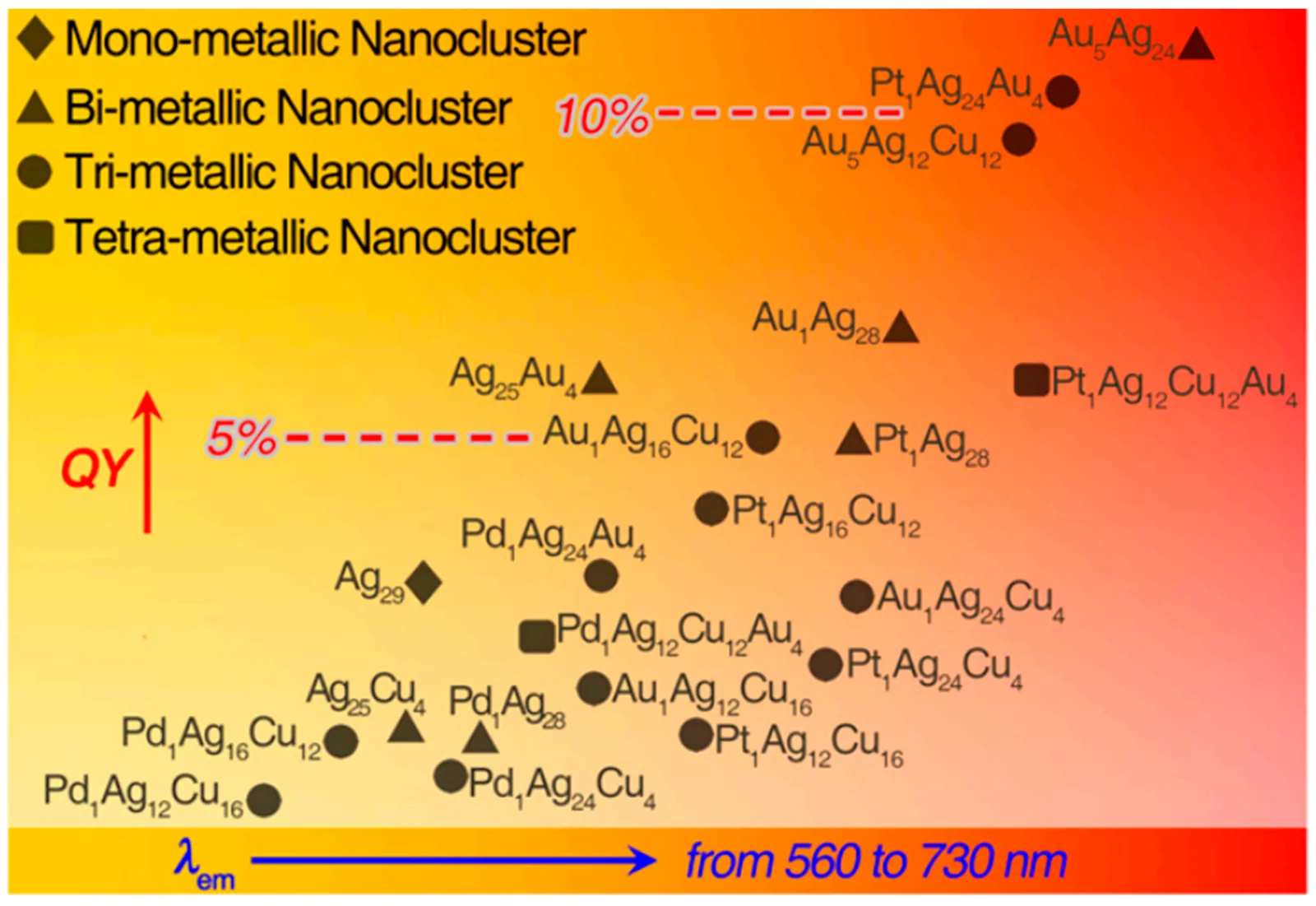
Comparison of luminescence properties of the M_29_ clusters (Reproduced under CC-BY 4.0 license http://creativecommons.org/licenses/by/4.0/ from ref. [19]).

In another study regarding the M_29_ template, a database of 21 NCs, ranging from monometallic to tetrametallic, was designed [19]. Both optical properties and structural stability were systematically examined, and synergistic effects were established. All the NCs exhibited fluorescence (Figure 7). The introduction of a Pd atom into the center of M_29_ was found to diminish the luminescence intensity while causing a hypsochromic shift of the emission. Conversely, when the central silver atom was replaced with either platinum or gold, the luminescence intensity improved significantly, accompanied by a bathochromic shift in the emission wavelength. Regarding the second shell, replacing Ag_12_ with Cu_12_ resulted in a hypsochromic shift of the emission wavelength and a slight reduction in the luminescence intensity. At the vertex positions, substituting silver atoms with gold not only caused a bathochromic shift in the emission but also markedly amplified the luminescence intensity. In contrast, replacing silver atoms at the vertices with copper led to the opposite effects. Among all the studied configurations, the Au_1_@Ag_12_@Ag_12_(S-Adm)_18_@(Au-PPh_3_)_4_ cluster demonstrated the highest fluorescence intensity and the longest emission wavelength, achieving a QY of ca. 12% and an emission peak at 715 nm. For comparison, the monometallic Ag_29_ cluster possessed an emission peak at ca. 620 nm and a QY < 5%. The authors noted that modification of the kernel or vertex metals of the M_29_ template with elements with higher electron affinity increased QY [19]. It seems interesting that in the two examples above, the highest QY is exhibited by clusters with the same stoichiometry—Au_5_Ag_24_. Again, this example demonstrates that site-specific alloying can enhance luminescence through changes in metal composition while preserving the overall structural framework of the parent cluster, highlighting the importance of electronic effects that can arise without major structural rearrangement.

Bootharaju et al. developed a ligand-exchange strategy to synthesize uniformly sized Pt-doped silver NCs denoted as Pt_1_Ag_28_. This was achieved through replacing the ligands in [Pt_2_Ag_23_Cl_7_(TPP)_10_] NCs with 1,3-benzenedithiol. The Pt dopant resided in the core of the Pt_1_Ag_28_ cluster, adjusting its electronic properties (the UV-Vis spectrum of Pt_1_Ag_28_ was slightly shifted to the UV region as compared to the monometallic Ag_29_) and significantly improving the luminescence intensity by 2.3 times. The ES lifetime exhibited a two-component behavior with 100 and 1800-ns lifetimes compared to 270 ns for the Ag_29_ NC [48]. The ES dynamics closely resembles that observed in the above example of AuAg NCs [6]. The authors noted that these changes might be attributed to the presence of surface-localized states proposed earlier [52]. It should be noted that the increase in QY correlates with the increase in the averaged luminescence lifetime, which suggests the suppression of nonradiative channels. Thus, in this case, both electronic-structure modification and suppression of nonradiative relaxation contribute to the luminescence enhancement.

A Pt_1_Ag_28_ NC stabilized by adamantane mercaptan and triphenylphosphine also showed excellent photoluminescence properties [49]. A Pt_1_Ag_28_(S-Adm)_18_(PPh_3_)_4_ NC exhibited a tetrahedral geometry, which was obtained by etching the alloy NCs with thiolate. The Pt_1_Ag_28_ core was obtained by reacting Pt_1_Ag_24_(SPhMe_2_)_18_ with thiolate and phosphine-type ligands. Remarkably, the Pt_1_Ag_28_ cluster demonstrated significantly improved luminescence properties with a QY of 4.9% and an emission maximum at ca. 672 nm. In contrast, both the precursor Pt_1_Ag_24_ NC and the Ag_29_ NC exhibited only low-intensity luminescence with a QY < 1%. When transiting from Ag_25_ to Pt_1_Ag_24_, the fluorescence lifetime showed a modest increase, rising from 1.1 μs to 1.9 μs. In contrast, for Ag_29_, the transition to Pt_1_Ag_28_ results in a shift from a single lifetime of 300 ns to dual lifetimes of 300 ns and 3.3 μs, along with a ca. 5-fold luminescence enhancement. Again, the ES dynamics look like in the examples above. Thus, the luminescence QY and lifetime of Pt_1_Ag_28_ are significantly increased in comparison with the monometallic Ag_29_ NC. This might indicate a significant influence of the Pt atom on nonradiative deactivation of the ES of the Pt_1_Ag_28_ NC. The authors proposed that the electronic structure of the Pt_1_Ag_28_ NC was changed, which might enhance the LMCT and increase the QY [49].

Not only silver but also copper metal atoms can enhance the luminescence of gold clusters. Gold−copper NCs can exhibit highly promising luminescent properties. Thus, a Cu_1_Au_38_ alloy NC was developed through introduction of a single Cu atom into the Au_38_(2-PET)_24_ cluster. The luminescence of the resulting Cu_1_Au_38_ adduct exhibited a two-fold enhancement when compared to the original Au_38_ NC [12]. However, the microscopic origin of this enhancement was not established in that study.

**Figure 8:**
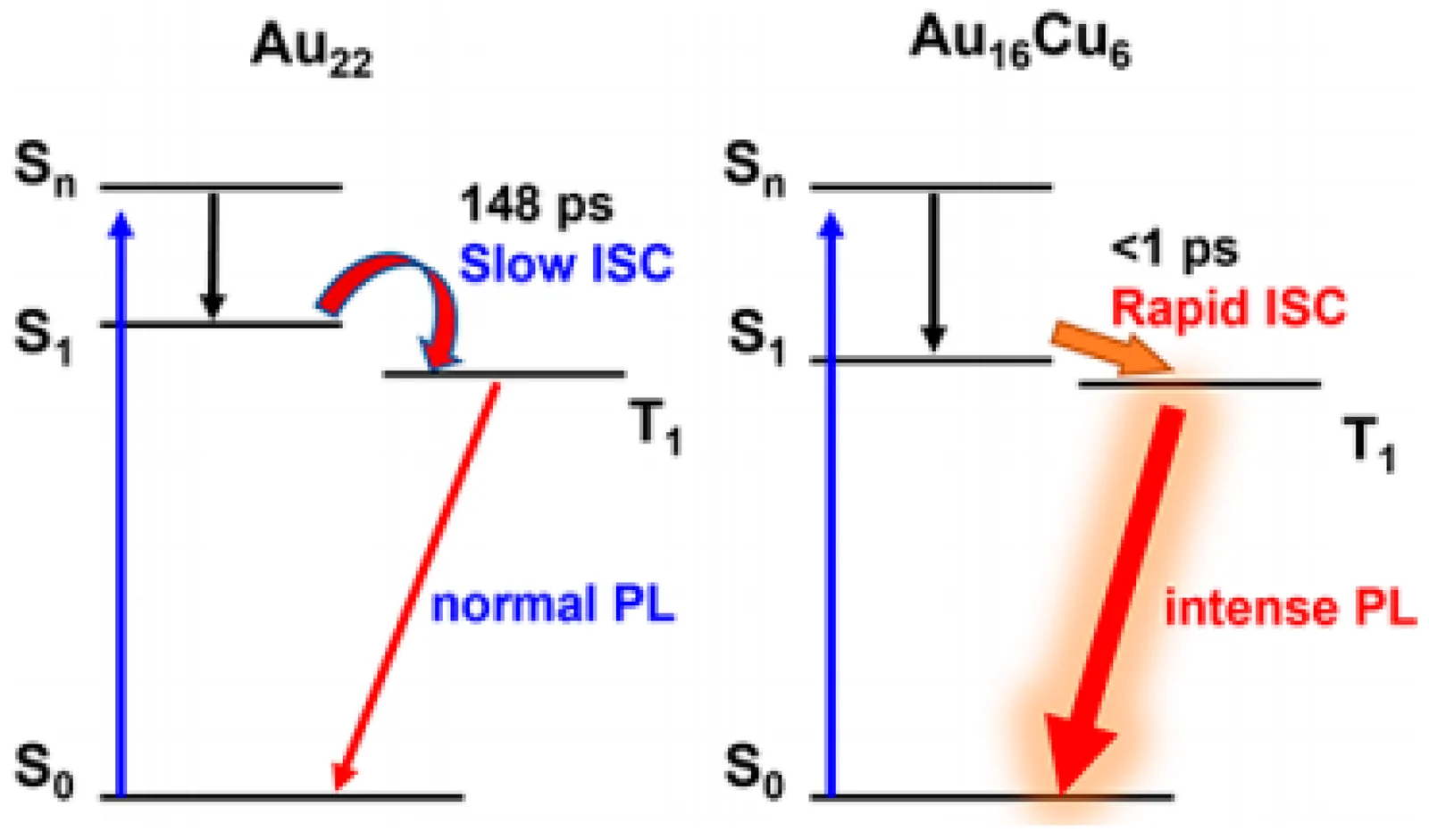
Schematic diagram of the excited-state relaxation pathways in Au_22_ and Au_16_Cu_6_. (Reproduced with permission from [27]. Copyright 2024 Science).

A dramatic enhancement of intersystem crossing has been recently observed for Cu_6_Au_16_ BNCs [27]. Although it is not thiolate-protected NC, we pay attention to it because of its exceptional luminescence properties. The photophysical properties of Au_22_(tBuPhC≡C)_18_ and its alloy counterpart, Cu_6_Au_16_(tBuPhC≡C)_18_ have been investigated in a joint theoretical and experimental study. It was observed that copper doping significantly influenced the optical behavior of the NC, leading to a ~60-fold reduction in nonradiative decay and a dramatic enhancement in the ISC rate by ca. 300 times: the ISC rate of 148 ps was observed in Au_22_, whereas Au_16_Cu_6_ exhibited a remarkably rapid ISC of ca. 0.5 ps (Figure 8). Due to this rapid ISC, the Cu_6_Au_16_ NC demonstrated exceptional luminescence QY reaching over 99% in de-aerated solution at room temperature. Similarly, in the Au_60_/Cu_x_Au_61−x_ pair [53], Cu insertion speeds up the ISC by about four times and turns an almost “dark” cluster into a NIR-II phosphorescent emitter.

And vice versa, the luminescence of copper NCs can be enhanced with gold atoms. The work of Silalahi et al. [50] presents a comprehensive series of two-electron M@Cu_12_ (M = Au, Ag) BNCs with a generalized composition [M@Cu_12_(dithiolate)_6_(C≡CPh)_4_]^+^, where the dithiolate ligands are systematically varied between dithiocarbamate and dithiophosphate/dithiophosphinate derivatives. All clusters have an M-centered Cu_12_ cuboctahedral core with idealized *T_d_* symmetry. These Au@Cu_12_ clusters demonstrate remarkable QYs of 23–55% in solution at room temperature, with the highest values (55%) observed for [Au@Cu_12_{S_2_P(O*^n^*Pr)_2_}_6_(C≡CPh)_4_]^+^ featuring chloride counteranions. Emission lifetimes in the microsecond time range indicate a phosphorescence-type emission. The QY follows the order Au@Cu_12_ > Ag@Cu_12_ ≫ Cu@Cu_12_. The Au-centered species exhibit superior photophysical properties compared to their Ag counterparts (QY of 0.58%), attributed to the enhanced spin-orbit coupling of gold, facilitating efficient ISC. Another example of the ISC enhancement is the AuCu_14_ NC [51]. The phosphorescence is 1.7-fold enhanced. The principal role of the central heavy Au atom in this system is confirmed by the decreasing QY upon replacement of the Au atom by Cl, suggesting a spin-orbit coupling effect. In addition, the luminescence lifetime is also increased by 1.6 times, indicating the suppression of the nonradiative channel. The decrease in k_nr_ in this case evidently plays a major role in the luminescence enhancement.

Table 1 highlights several important trends in thiolate-protected polymetallic NCs. First, large enhancements in luminescence are not restricted to a particular metal pair: Ag incorporation into Au clusters, Au or Pt incorporation into Ag clusters, and Cu incorporation into Au clusters can all produce substantial changes in emission. Second, the magnitude of the enhancement varies widely, from modest increases to more than two orders of magnitude, indicating that metal incorporation alone does not determine the optical response. The most informative examples are those in which the atomic framework remains conserved or the excited-state dynamics have been characterized. These studies show that enhanced emission can arise from different processes, including suppression of nonradiative relaxation, modification of radiative transitions, and enhanced intersystem crossing.

Resuming this section, a large body of work has been devoted to studying the influence of alloying on the emission properties of metal nanoclusters (MNCs). The underlying problem is the low photoluminescence QY for most of the clusters, which limits their applications in optics and bioimaging. Many researchers employ a set of steady-state and ultrafast spectroscopic techniques, as well as quantum-chemical calculations, to trace at the atomic level how metal doping, substitution, or binding at the surface reorganizes the bands, excited states, and relaxation channels. At present, it is difficult to form a coherent picture of the relationship between structure, electronic configuration, ES dynamics and luminescence. Nevertheless, a number of patterns can be identified.

ES dynamics is a key to understanding the origin of the luminescence QY enhancement in polymetallic NCs. The *QY* is basically determined by the competition between the radiative (*k_r_*) and nonradiative (*k_nr_*) decay rates:

(1) $$QY=\frac{k_{r}}{k_{r}+k_{nr}}$$

The *QY* is directly proportional to the luminescence lifetime (*τ*):

(2) $$QY=\tau k_{r}$$

Thus, in order to enhance *QY*, one needs either to increase *k_r_* or decrease *k_nr_*. A decrease in *k_nr_* evidently means that nonradiative decay channels are suppressed. In this case, some vibrational or rotational modes are frozen. Suppression of the nonradiative channel is the most common reason for luminescence enhancement. If luminescence originates from a triplet state, the competition between *k_nr_* and ISC decay rates is also essential.

**Figure 9:**
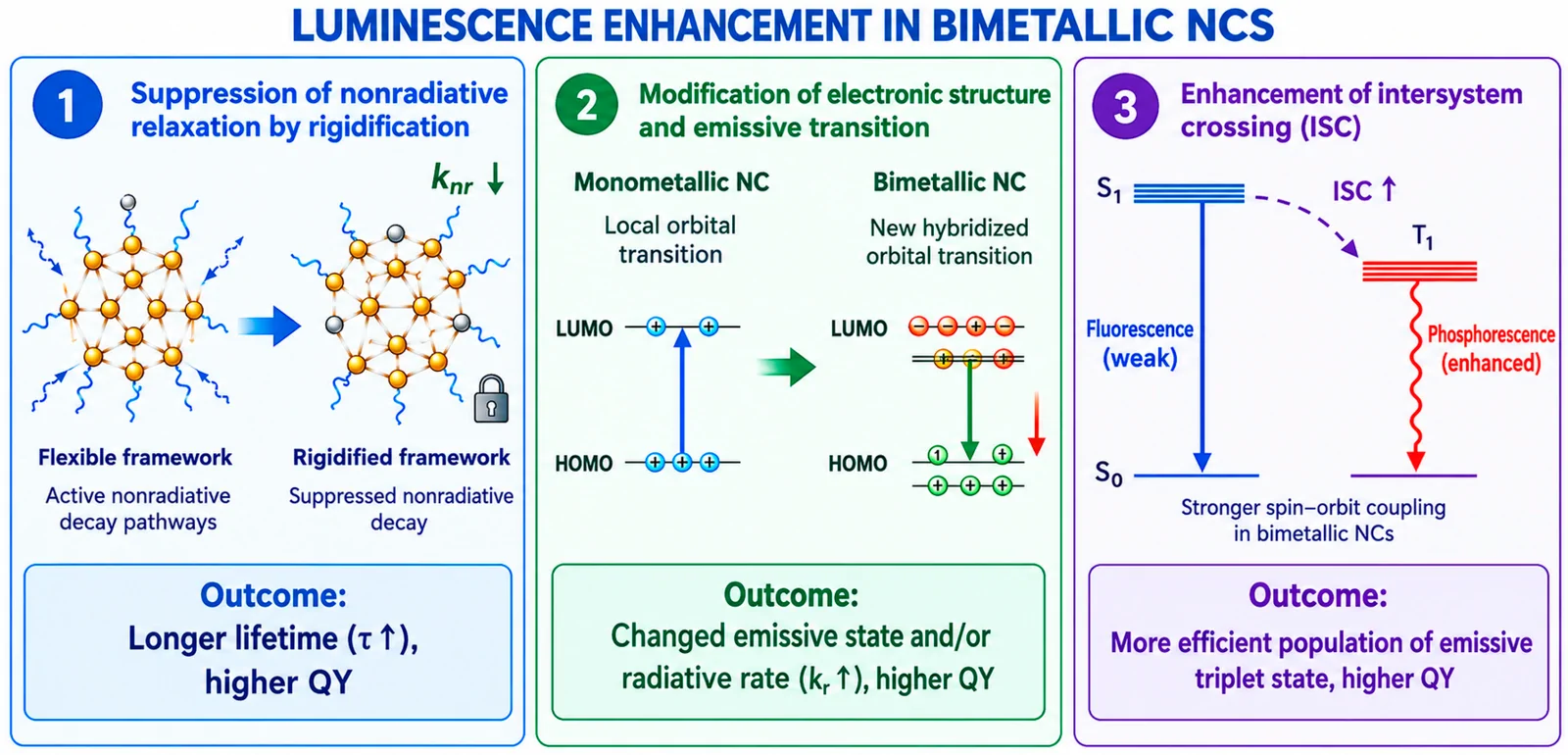
Main factors of luminescence enhancement in bimetallic NCs.

Three main factors of luminescence enhancement can be highlighted (Figure 9), but these factors are not necessarily mutually exclusive mechanisms. Incorporation of a second metal can simultaneously modify the cluster geometry, metal–ligand interactions, electronic structure, vibrational dynamics, spin–orbit coupling, and excited-state relaxation pathways. As a result, structural and electronic effects can be intrinsically coupled. In some cases, the available experimental data allow one to identify a dominant microscopic origin of the observed luminescence enhancement.

One contribution is rigidification of the cluster core and/or ligand shell. Thereby, some vibrational modes are frozen and the nonradiative rate constant *k_nr_* can be decreased. At the same time, the ES lifetime is increased. As a result, the QY is increased (Formulas (1) and (2)). For instance, incorporation of silver ions into the framework of the GSH-based Au NCs makes the ligand shell more rigid and suppresses nonradiative channels [40]. The ES dynamics of the silver Ag_29_ cluster also indicate a decrease in the rate of nonradiative transition in the case of Au doping [6]. The luminescence QY along with the lifetime of the Pt_1_Ag_28_ cluster are significantly increased in comparison with the monometallic Ag_29_ NC, which also suggests a significant decrease in the rate of nonradiative deactivation of the ES of the Pt_1_Ag_28_ NC.

A second contribution is the modification of the electronic states and of the radiative transition. Changes in metal composition can alter the spatial distribution and character of the frontier orbitals, transition energies, oscillator strengths, and charge-transfer character of the emissive state, potentially increasing *k_r_*. The rod-shaped Au_12_Ag_13_ cluster provides a particularly instructive example: Ag incorporation changes the character of the HOMO–LUMO transition, while the alloy also exhibits changes in structural relaxation and nonradiative decay relative to Au_25_ [44]. Thus, the increased *k_r_* in this system cannot necessarily be separated from the structural changes induced by alloying. Similar considerations apply to BSA-AuAg and DNA-AgCu clusters, for which increases in QY occur despite shorter fluorescence lifetimes, suggesting that an increase in the radiative rate may contribute substantially to the enhancement [54,55].

A third contribution is enhanced intersystem crossing. Incorporation of a heavier or otherwise electronically influential metal can modify spin–orbit coupling and facilitate population of emissive triplet states. This effect has been demonstrated particularly clearly for Cu_6_Au_16_ [27] and AuCu12 clusters [44]. However, enhanced ISC should also be considered as part of a coupled excited-state process rather than as an independent effect of the dopant. The same metal incorporation that modifies spin–orbit coupling can also change the electronic structure, structural rigidity, and nonradiative decay pathways of the cluster. In Cu_6_Au_16_, for example, the exceptional phosphorescence QY is associated not only with accelerated ISC but also with a strong reduction in nonradiative decay [27].

A further complication in assigning the origin of luminescence enhancement is that the polymetallic and monometallic species used for comparison are not always structurally equivalent. Introduction of a second metal may change not only the elemental composition but also the nuclearity, atomic arrangement, surface motifs, oxidation state, ligand coordination, charge state, or aggregation behavior of the NC. Consequently, an observed increase in QY or emission intensity cannot automatically be attributed to the identity of the second metal itself. The most informative comparisons are those in which the monometallic and polymetallic NCs retain the same or closely related nuclearity and ligand environment, allowing the effect of metal substitution to be more clearly isolated. Even in such cases, however, local structural rearrangements and changes in metal–ligand interactions may accompany alloying. One should therefore use the terms “metal-composition effect” or “bimetallic effect” cautiously and distinguish direct evidence for the role of the incorporated metal from effects that may arise indirectly through structural or electronic reorganization. This distinction is particularly important for biopolymer-protected NCs, for which atomic structures are often unavailable and changes in cluster composition may also alter the ligand environment and supramolecular organization.

## 3. Bimetallic NCs Stabilized by Biomolecules and Their Applications

One of the advantages of NCs templated by biomolecules is their good biocompatibility. Another interesting feature is the ability of specific interactions with other biomolecules. These features of NCs templated by biopolymers explain the special interest in these clusters. The doping of various metals can modulate their spectral properties and increase the luminescence QY. The QY of BNCs is often higher than that of monometallic ones [55,56,57,58,59,60,61].

The composition and architecture of BNCs are varied by combining different metal pairs (Au-Ag, Ce-Au, Au-Cu, Zn-Cu, Cu-Ag, Ag-Pd, Ag-Pt, Au-Ni, Au-Pt, Co-Au) and stabilizing agents. The predominant synthesis method for such alloyed systems is the co-reduction strategy. Bovine serum albumin (BSA) is the most common ligand of all proteins. However, several studies also report the successful use of lysozyme [61,62,63]. Such matrices can simultaneously perform both reducing and stabilizing functions.

AuAg is the most frequent type of BNCs. In one of the first works, Pradeep’s group reported on the synthesis of BSA-AuAg alloys [64]. The luminescence enhancement factor of most synthesized BSA-AuAg clusters is in the range of 0.4–2.7, while the QY is typically in the range of 3–17% (Table 2). Note that in some cases the luminescence of AuAg clusters can be weaker than that of monometallic clusters. For comparison, the QY of the BSA-Au NCs was reported to be ca.8% [65]. An exceptional case is the QY of 42% reported in [54]. It should be noted that in that case the Au:Ag ratio is 2.3: 2.0, while under other synthesis conditions it is typically 6:1. The authors do not discuss the possible mechanisms of the luminescence enhancement. However, based on the data reported in that paper, we can draw several conclusions. The NCs exhibited an average luminescence lifetime of 14.2 ns, which is markedly decreased, while the QY is increased, compared to the Au NCs. From expression (2) it follows that *k_r_* is increased, indicating an increase in the transition dipole moment. Therefore, it can be proposed that the electronic structure of the cluster is changed. As the luminescence lifetime is typical of Ag NCs rather than Au NCs, it could indicate a shift in the nature of the HOMO-LUMO transition toward orbitals localized on the Ag atoms. A similar conclusion about an increase in *k_r_* was reported in [66], based on the ES dynamics of AuAg clusters templated by BSA.

The QY for Lyz_AuAgNCs was reported to be 7.5%, compared to 1.5% for Lyz_AuNCs [61]. Among other metal pairs were CeAu, AuCu, ZnCu, AuNi, and AuPt. The reported values of EF and QY lie in the same range as most values for AuAg NCs (Table 2). The possible mechanisms of the apparent luminescence enhancement are not discussed in most cases.

In addition to proteins, thiolate ligands such as GSH [60,62,67], a peptide [57] and penicillamine [68] are used. The EF values and the reference QYs for the corresponding monometallic NCs in most studies are not reported. The QYs of BNCs are typically in about the same range as those for the protein-templated NCs (Table 2). The highest QY of ca. 16% was reported for AuAg-GSH NCs in one of the first papers on BNCs stabilized by GSH [69]. The fluorescence intensity of the AuAg-GSH NCs was 4–5 times higher than that of the AuGSH NCs. Possible mechanisms of the luminescence enhancement were not discussed. The AuAg-GSH NCs exhibited high chemical stability in the pH range of 3–10 and high photostability. A high two-photon excitation efficiency was also observed.

Nucleosides [70], nucleotides [71,72], and DNA sequences [55,59,73,74,75,76] represent one more class of biological templates, where the bases serve as a ligand for forming BNCs with emission properties that are extremely sensitive to the DNA length and sequence. It is not surprising, since it has been demonstrated in a large number of works on monometallic silver NCs on DNA matrices (see, for example, [77,78,79]). As to BNCs, Chen et al. reported that the sequence 5′-CCCTTAATCCCC-3′ enables the production of DNA-AuAgNCs possessing stronger fluorescence compared to other sequences (5′-CCCCCCCCCCCC-3′ and 5′-CCCTCTTAACCC-3′) [59]. Furthermore, in the work by Li et al., five DNA strands (A12, C12, T12, G4-ATAT-G4, C4-ATAT-C4) were used as templates for the formation of AuAgNCs, and only one of them was suitable for forming fluorescent AuAgNCs [74]. It is worth noting that there are very few works on BNCs on DNA compared to works devoted to monometallic clusters.

DNA bases can serve as a model system for long oligonucleotides. The adenosine monophosphate (AMP)-protected AuAg BNCs have been synthesized using a hydrothermal synthesis method [80]. A less common but valuable alternative to co-reduction is doping with metal ions, where a pre-formed monometallic NC undergoes the addition of ions of another metal. The application of this method in systems such as cytidine-stabilized gold NCs can lead to an immediate 25-fold enhancement of fluorescence upon the addition of silver [70]. The authors noted that interaction of silver ions or reduced silver species with C-AuNCs is possible, causing the fluorescence enhancement. It is worth mentioning that a similar picture was discussed in the previous section in the case of GSH-Au NCs. By analogy, it is reasonable to suggest that the aggregation-induced emission (AIE) effect due to forming C-Ag(I)-C bonds probably takes place.

The luminescence of most studied BNCs stabilized by oligonucleotides [59,73,74,75,76] does not exhibit significant enhancement or QY. Thus, in [73], Ag-Pt, Ag-Pd, and Ag-Au NCs stabilized with different DNA sequences were studied. It was noted that the fluorescence emission of the DNA-AgNCs decreased with increasing concentrations of metal dopants. The evolution of the absorption, luminescence excitation, emission and XPS spectra of the DNA-Au–Ag NCs was studied in dependence on the Ag:Au ratio in work [75]. The authors concluded that the Au–Ag NCs formation could be decomposed into two steps: the formation of Ag NCs followed by oxidization of Ag NCs by gold in the second step. The emission of DNA-Au–Ag NCs demonstrated a presence of microsecond components in addition to the nanosecond components typical for Ag emission. It should be noted that the microsecond decay is typical for Au NCs.

In the case of DNA-AgCu NCs, a significant fluorescence enhancement of ~5 times was obtained compared to DNA–Ag NCs. The BNCs were reported to have QY = 51.2% [55]. The DNA sequence was a 12-mer 5′-CCCTTAATCCCC-3′. For comparison, the QY of Ag NCs stabilized by this sequence was reported to be 11.5% in that work and 38% in [77]. It is interesting to note that the average fluorescence lifetime of ca. 2 ns [81] of the AgCu NC appears to be two times less than that of the 4 ns measured earlier [77] for the corresponding monometallic Ag NC. Normally, the QY is proportional to the lifetime of the ES (Equation (2)). The discrepancy between the rise in the QY and the drop in the lifetime can be rationalized in the following way. Similar to the above cases of BSA-templated AuAg NCs [55], *k_r_* evidently increases upon doping with Cu. This suggests that the nature of the HOMO-LUMO transition in the AgCu NCs changes compared to Ag NC. Additionally, structural changes may contribute to the increase in the QY.

A distinctive optical feature of BNCs is their tunable luminescence [54,75]. Simply varying the molar ratio of the two metal precursors during synthesis allows for the regulation of fluorescence emission across a broad spectral range. This is clearly demonstrated in the work by Sannigrahi et al., where adjusting the Au:Ag ratio enabled a shift in the emission peak from 680 nm to 815 nm, providing access to the NIR region, which is ideal for tissue bioimaging due to reduced light scattering and autofluorescence. [54]. In most systems, either a blue shift in emission is observed when Ag is added to AuNCs [56,82,83,84], or a red shift occurs when Au is added to AgNCs [85].

The stability of NCs is a critical factor for practical applications, and here BNCs demonstrate a decisive advantage. They exhibit exceptional resistance to a variety of challenging conditions that often destroy MNCs: extreme pH values, environments with high ionic strength, prolonged photoirradiation, and the presence of oxidizing agents [58,59,63,74,75,83,85,86]. A clear example is stability in saline solutions (200 mM NaCl): DNA_Au-AgNCs retained 20% of fluorescence, while DNA_AgNCs lost 99% of the signal under the same conditions, making them virtually useless for physiological diagnostics [74].

Another example of exceptionally high stability is described in the work by Sun et al. The authors investigated the fluorescence stability of NCs against potential interferents such as ions of the most common metals, anions, biothiols, amino acids, H_2_O_2_, FBS, and RPMI 1640. Under neutral conditions, these interferents did not cause a noticeable effect on the fluorescence intensity. The NCs also demonstrated excellent photostability after several hours of measurements [83].

Such high stability of BNCs also prevents undesirable aggregation. Although both MNCs and BNCs can, in certain cases, demonstrate the AIE effect, leading to luminescence enhancement [71,72]. The AIE effect is a phenomenon where molecular aggregation results in the emergence or enhancement of luminescence. The AIE process is primarily driven by intermolecular interactions between ligands causing aggregation while the cluster core remains intact. In the work by Zhang et al., the AIE effect is observed upon the addition of aluminum ions to AMP-stabilized gold-silver BNCs [71]. When strongly positively charged aluminum ions are added, electrostatic interaction occurs between Al^3+^ and the negatively charged phosphate groups of AMP. This is confirmed by zeta potential measurements: its value changes from negative to positive, proving the binding of aluminum cations to the surface. Thus, Al^3+^ acts as a bridge, cross-linking individual NCs. As a result, large spherical aggregates greater than 50 nm are formed, whereas the average diameter of the initial NCs is 2 nm. The cross-linking between AuAgNC@AMP and Al(III) limits the vibration and rotation of functional groups of AMP on the surface, which consequently enhances the emission by reducing the nonradiative decay [71].

The synergistic effect of the two metals directly enhances the analytical performance of sensor applications. Sensitivity, measured by the limit of detection (LOD), is often several times higher for BNCs [59,75,76,82]. A sensor based on BSA-AuAg NCs demonstrated a 6.3 times higher sensitivity for Hg^2+^ detection compared to an equivalent sensor based on BSA-Au NCs [82]. Similarly, DNA-AuAg NCs achieved a remarkably low LOD of 1.64 nM for Hg^2+^, significantly surpassing the LOD of DNA-Ag NCs, which were 29.2 and 35.1 nM for different emission peaks [75]. The improvement in analytical performance is not limited to sensitivity alone but also includes selectivity. The unique structure of BNCs allows the fluorescent response to be specific to a particular analyte, minimizing interference from other metal ions. For example, the Au(I) protective shell in some BNCs has been found to prevent anti-galvanic reduction, thereby eliminating interference from copper ions, a common problem for Ag-based sensors [75].

**Figure 10:**
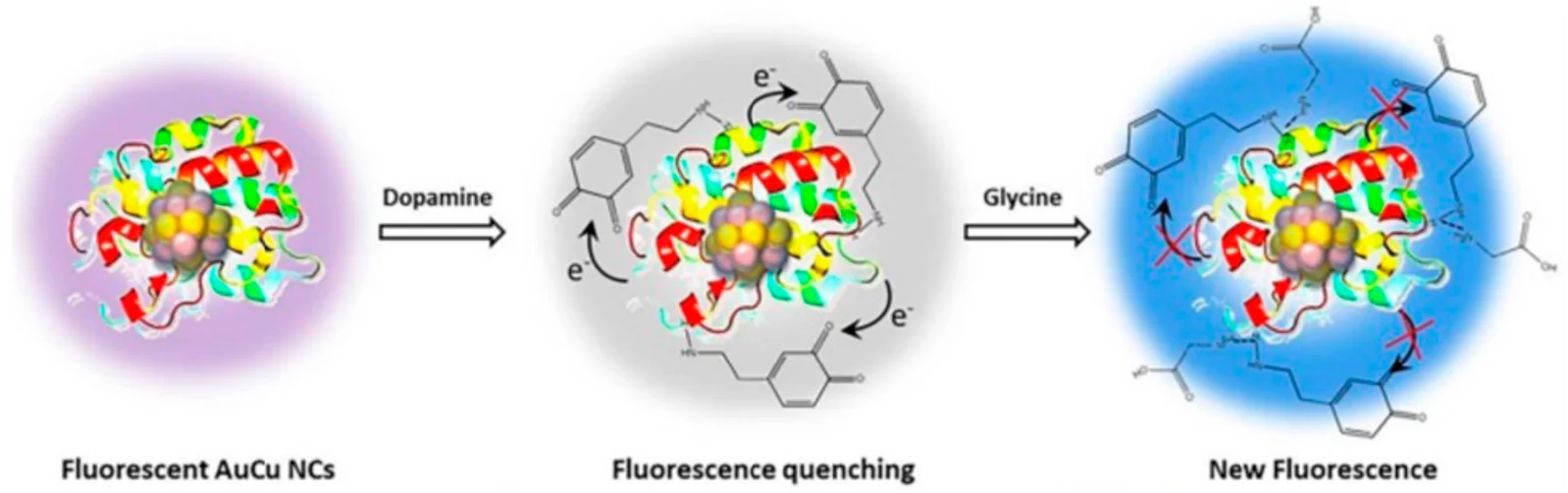
Working principle of dopamine-modified AuCu NCs for glycine-selective sensing. (Reproduced with permission from [58]. Copyright 2020 American Chemical Society).

In sensing applications, BNCs are employed not only in classic luminescence quenching/enhancement sensors but also in more sophisticated schemes such as “on-off” strategies, emission spectrum shifts, or the appearance of new peaks. For example, Chen et al. developed a straightforward strategy for fabricating dopamine-modified AuCu NCs as a charge-transfer-based biosensor for highly sensitive glycine detection. The BSA-stabilized AuCu NCs exhibited a fluorescence maximum at 400 nm. Owing to the high affinity of BSA for dopamine, the NC surface was modified with dopamine without complex chemical reactions, resulting in fluorescence quenching via charge transfer. When the biosensor interacts with glycine, hydrogen bonding with oxidized dopamine inhibits the charge transfer process, leading to the emergence of a new emission peak at 475 nm (Figure 10) [58].

Su et al. reported the use of a combination of DNA-CuAg NCs and 3-mercaptopropionic acid (MPA) for the detection of Cu^2+^ ions. Thiol compounds, when binding to the NCs, weaken the interaction between the DNA templates and the metal clusters, resulting in fluorescence quenching. The fluorescence of the NCs is quenched in the presence of MPA and then restored upon the addition of copper. The presence of Cu^2+^ ions leads to the oxidation of MPA to form a disulfide compound. Consequently, the strength of the interaction between the thiols and the NCs decreases, thereby suppressing fluorescence quenching and restoring the fluorescence [81].

**Figure 11:**
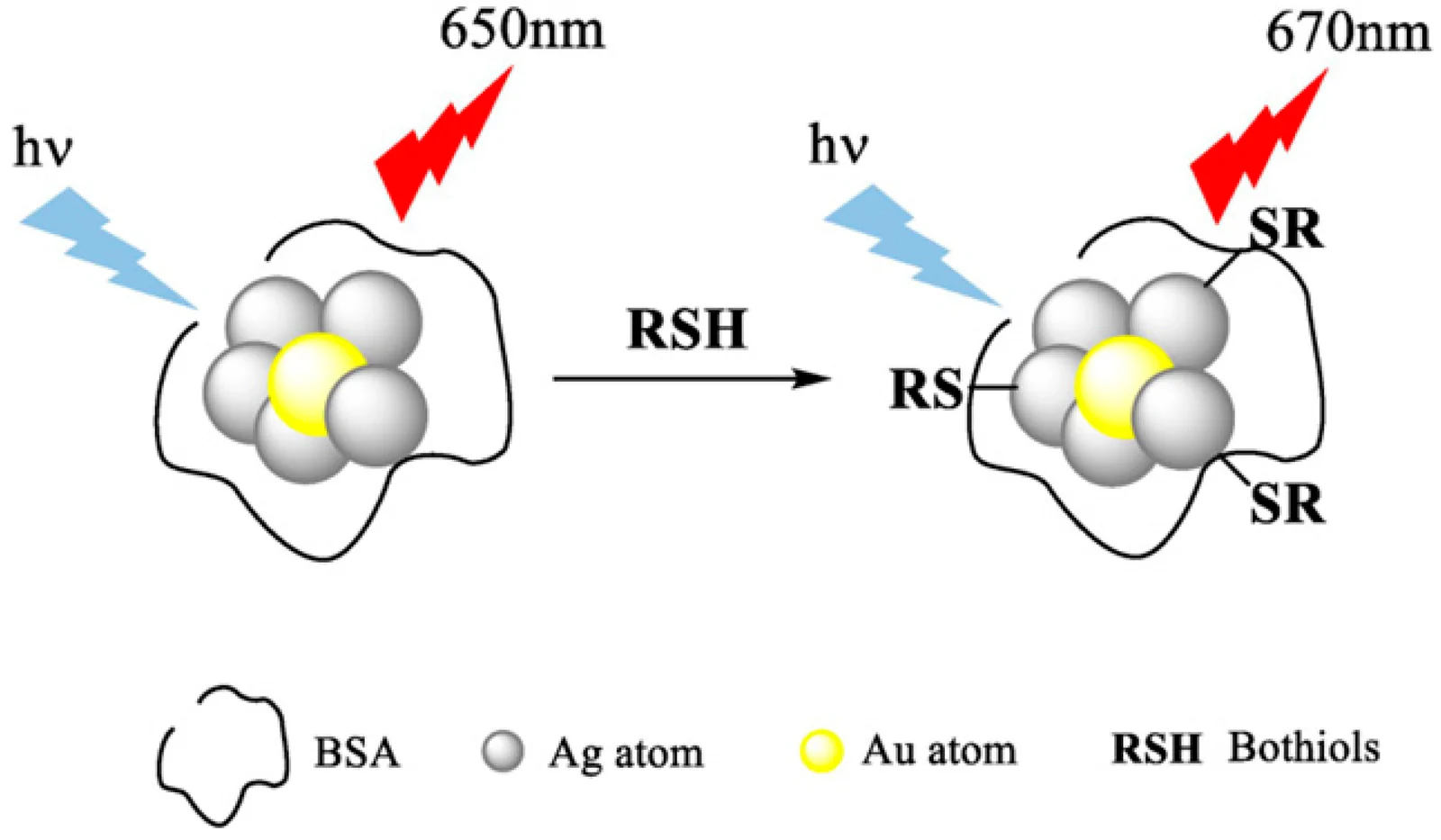
Schematic representation of Cys-induced red shift in AuAg NCs fluorescence. (Reproduced with permission from [85]. Copyright 2019 Elsevier).

DNA-Cu/Ag NCs have also been used for the detection of adenosine [87], microRNA [88], and sulfide [89]. Feng et al. developed a method for cysteine detection using BSA-stabilized AuAg NCs based on a shift in the maximum emission wavelength. Cysteine binds to AuAg NCs via coordination between metal and sulfur atoms, resulting in a change in the emission wavelength. It is reported that the luminescence peak shift offers greater accuracy than intensity variations, as the latter can easily be influenced by environmental interferents and/or other factors (Figure 11) [85].

As can be seen, the application scope of sensors based on BNCs extends far beyond the detection of metal ions (Hg^2+^, Cu^2+^, Pb^2+^, Fe^3+^, Cd^2+^, Cr^3+^) [54,56,68,75,76,81,82,90,91,92] and sulfur-containing species [59,89]. BNCs serve as effective probes for a range of biomolecules such as GSH [84], cysteine [71,85], glycine [58], glucose [62], folic acid [72,93], uric acid [62], H_2_O_2_ [62], ascorbic acid [63], single-stranded DNA-binding protein (SSB) [55], adenosine [87], and nucleic acids [74,88,94]. They also constitute powerful platforms for enzyme detection [63,95]. Approaches are also being developed for the detection of antibiotics, in particular oxytetracycline, which can be detected and visualized using appropriate BNCs [96].

**Figure 12:**
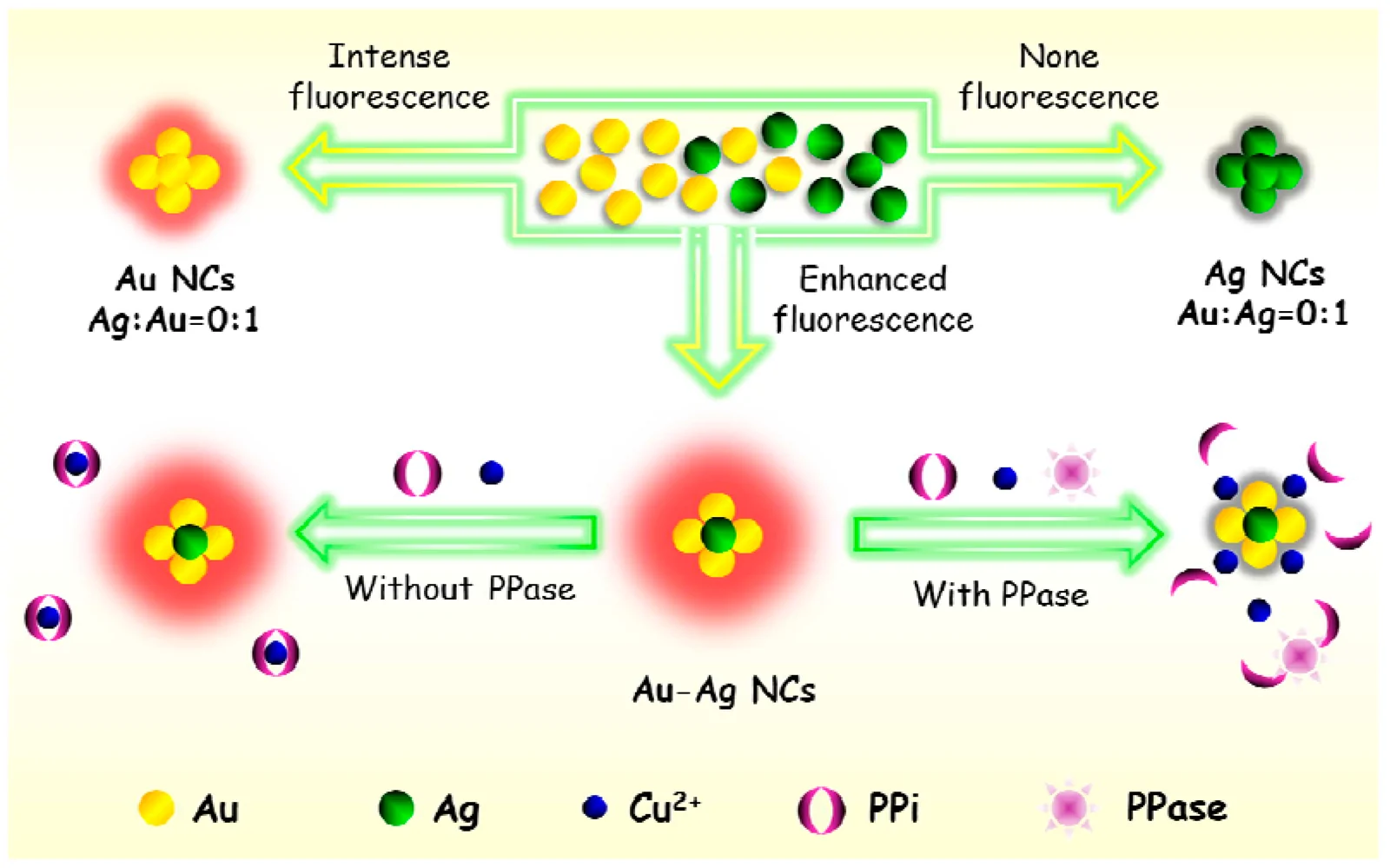
Schematic representation of the Au-Ag BNCs-based fluorescent probes used to quantify inorganic pyrophosphatase (PPase) activity. The mechanism couples the enzyme-triggered hydrolysis of pyrophosphate (PPi) with the dissociation of copper ions (Cu^2+^) from the Cu^2+^-PPi complexes. (Reproduced with permission from [97]. Copyright 2016 American Chemical Society).

A strategy for detecting inorganic pyrophosphatase (PPase) activity, based on a PPase-regulated competitive reaction involving Cu^2+^, pyrophosphate ion (PPi), and BSA-stabilized AuAg NCs, was reported (Figure 12). Cu^2+^ was used as a fluorescence quencher for BSA-AuAg NCs, while PPi served as the hydrolytic substrate for PPase. In the absence of the target PPase, free copper ions initially formed complexes with PPi via coordination chemistry, thus preserving the natural fluorescence intensity of the NCs. When the target PPase was introduced into the system, it hydrolyzed PPi into phosphates, thereby releasing Cu^2+^ ions from the complexes. Consequently, the freed copper ions readily quenched the fluorescent signal of the clusters [97].

The functional versatility of BNCs is further evidenced by their use as ratiometric sensors for pH and temperature [83,86]. BNCs have been successfully employed as highly sensitive fluorescent thermometers. The fluorescence intensity decreases linearly within the range of 10 to 50 °C. A key advantage is their high temperature resolution, ranging from 0.1 to 0.5 °C, enabling the detection of even minor fluctuations. The thermometer demonstrates excellent reversibility and stability over multiple heating and cooling cycles. Comparison with other fluorescent thermometry systems confirmed that the resolution of NCs is comparable to or superior to that of state-of-the-art fluorescent thermometers [83].

Several studies have demonstrated the use of BNCs as chemiluminescence [67] and electrochemiluminescence emitters [94]. BNCs also find applications in theranostics, for example, in imaging and gene delivery [57,60,61,98]. These two applications were combined in the work by Dutta et al. [98], where a cationic BSA nanoparticle system incorporating AuAg NCs was developed for the delivery of a therapeutic suicide gene into HeLa cancer cells. The luminescence of the NCs enables visualization of the delivery process and intracellular localization.

The catalytic activity of NCs is also significantly enhanced in bimetallic systems. DNA-templated Ag-Pd, Ag-Au, and Ag-Pt BNCs demonstrated substantially improved catalytic activity in reactions such as the reduction of 4-nitrophenol compared to DNA-AgNCs. The rate constant increased by 2.4 times with Pd and Pt doping [73].

**Table 2:** Luminescent ligand-bimetal NCs for biosensing applications.

Metal Core	Ligand	Method of Synthesis	QY Enhancement Factor (EF) QY Proposed Mechanism	Applications Optical Features	Reference
Au-Ag	BSA	Mixing of the Pre-formed BSA-Au and BSA-Ag clusters	EF = 0.4 relative to BSA-Au QY = 2.8%	–	[64]
Au–Ag	BSA	Co-reduction	~2× luminescence intensity vs. Au NCs QY = 17.6%	Folic acid detection by luminescence quenching	[93]
Au-Ag	BSA	Co-reduction	EF = 1.2 QY = 9.6%	Cu^2+^ detection	[65]
Au-Ag	BSA	Co-reduction	EF ~ 2 QY = 10.5%	Hg^2+^ and Cu^2+^ detection by luminescence quenching	[56]
Au-Ag	BSA	Co-reduction	EF ~ 5 (relative to the known value of 8% for Au-BSA NCs [65]) QY = 42%. Proposed mechanism: increasing k_r_, changing the nature of the HOMO-LUMO transition [this review]	Pb^2+^ detection by luminescence enhancement. Fluorescence emission can be tuned from 680 nm to 815 nm by varying the Au:Ag molar ratio.	[54]
Au-Ag	BSA	Co-reduction	EF = N/A QY = 6.8%.	Hg^2+^ detection by luminescence quenching. The sensitivity of the sensor based on the AuAg NCs is 6.3 times higher than that based on the AuNCs. The emission maximum of the AuAg NCs is blue-shifted by 80 nm to 577 nm compared to that of the Au NCs. The prepared BSA-AuAg NCs have a good light stability	[82]
Au-Ag	BSA	Co-reduction	EF ~ 1 QY = 3.4%	Fluorescent thermometer, ratiometric pH sensor. Emission peak is blue-shifted by 60-nm to 570 nm compared to Au NCs. Highly biochemical stability toward various pH values, ions, biothiols, H_2_O_2_, fetal bovine serum, RPMI1640, aminoacids, and photoillumination	[83]
Au-Ag	BSA	Co-reduction	EF = N/A QY = N/A	Cys detection by red-shift luminescence. Biothiol detection. The AuAg NCs provide improved stability (resist oxidation) and a greater red-shift response to Cys than Ag NCs	[85]
Au-Ag	BSA	Co-reduction	~2.7× luminescence intensity vs. Au NCs QY = N/A	Sensor for GSH detection. Emission peak is blue-shifted by 40-nm to 640 nm compared to Au NCs.	[84]
Au-Ag	BSA	Co-reduction	~2× luminescence intensity vs. Au NCs QY = N/A	Sensing probes toward inorganic pyrophosphatase activity with the help of Cu^2+^ and inorganic pyrophosphate ion by quenching	[97]
Au-Ag	BSA (cationic)	Co-reduction	EF = N/A QY = 11.7% for the cationic BSA-Au-Ag NCs and 8.8% for the final pDNA-loaded composite nanoparticles	Gene delivery vector for HeLa cancer cells, bioimaging. Excellent photostability	[98]
Au-Ag	BSA Lyz	Co-reduction	EF = 1.7 for BSA-AuAg QY = 2.5% EF = 4.8 for LYS-AuAg QY = 7.5%	BNCs possess higher colloidal stability than the monometallic gold NCs stabilized by the same protein	[61]
Au-Ag	BSA	Co-reduction	EF = N/A QY = N/A	Biosensor for miRNA-21 detection. Early lung cancer screening. Electrochemiluminescence.	[94]
Au-Ag	BSA	Co-reduction	EF = 3.2 QY = 18% Proposed mechanism: increasing k_r_, changing electronic structure	Intracellular ratiometric thermometry.	[66]
Ce-Au	BSA	Co-reduction	EF = N/A QY = 8.7% (410 nm) QY = 5.8% (650 nm)	Ratiometric pH probes	[86]
Au-Cu	BSA	Co-reduction	EF ~2.5 relative to Cu NCs QY = 10.2%	Detection of glycine by emergence of a new emission peak	[58]
Zn-Cu	BSA	Co-reduction	EF = N/A QY = N/A	H_2_O_2_ detection by enhancement of chemiluminescence	[99]
Au-Ni	BSA	Co-reduction	EF = N/A QY = N/A	Cd^2+^ detection by luminescence enhancement and Hg^2+^ detection by quenching	[92]
Au-Pt	BSA	Co-reduction	EF ~ 1 QY = N/A	Hg^2+^ and Cys (off-on strategy) detection	[100]
Au-Ag	Lys	Co-reduction	EF = N/A QY = 4.5%	Detection of ascorbic acid and acid phosphatase by luminescence enhancement. Excellent stability under various pH and ionic strength conditions.	[63]
Au-Ag	Peptide	Co-reduction	EF = 4 QY = 6.5%	Targeting the lysosomes and monitoring hypochlorite in living cells	[57]
Au-Ag	GSH	Co-reduction	EF = 4–5 QY = 16% Proposed mechanism: both increasing k_r_ and decreasing k_nr_ [this review]	Excellent stability in the pH range 3–10; high photostability; large two-photon excitation efficiency	[69]
Au-Ag	GSH Lys	Co-reduction	EF = N/A QY = 18%	Detection of glucose and uric acid. Monitoring the fluctuation of the OH level. Detection of H_2_O_2_ based on the Fenton reaction. Good stability. The performance of this probe for H_2_O_2_ sensing was comparable with other Ag or Au NCs and especially improved in the linear range	[62]
Au-Ag	GSH	Co-reduction	EF = N/A QY = 13.0%	Nonlinear optical imaging of biological samples. Exhibits two-photon luminescence and second-harmonic generation under the NIR excitation. Optical properties are retained in a solid DNA matrix	[60]
Co-Au	GSH	Co-reduction	EF = N/A QY = N/A	Chemiluminescence sensor for determination of H_2_O_2_ The chemiluminescence intensity was much higher than that of GSH- AuNCs and GSH-CoNCs	[67]
Au-Ag	Chondroitin sulfate	Co-reduction	EF = N/A QY = 1.4%	“Turn-on-off-on” sensing platform for hyaluronidase detection	[95]
Cu-Au	Penicillamine	Co-reduction	EF = N/A QY = 3.5%	Fe^3+^ detection by fluorescence quenching	[68]
Au-Ag	Adenosine monophosphate	Hydrothermal synthesis method	EF = N/A QY = 8.5%	Bioimaging of HeLa cells	[80]
Au-Ag	Adenosine monophosphate	Hydrothermal synthesis method	EF = N/A QY = 8.5% The fluorescence of AMP-AuAgNCs was 3-fold enhanced upon assembly with Al(III), which was attributed to the AIE property ([71]).	Cysteine detection by fluorescence quenching	[71]
Au-Ag	Cytidine	Doping with Ag(I) ions	~25× luminescence intensity vs. Au NCs QY ∼ 9% Proposed mechanism: Ag^0^ or Ag^+^ interact with C-Au NCs	–	[70]
Au-Ag	Adenosine monophosphate	Co-reduction	EF = N/A QY = 7.3%	Folic acid detection by fluorescence quenching	[72]
Ag-Pd Ag-Au Ag-Pt	DNA	Galvanic replacement	EF < 1 QY = N/A	Reduction of 4-nitrophenol Catalytic activities of the BNCs are greatly improved compared to DNA-AgNCs	[73]
Au-Ag	DNA: 5′-CCC TTA ATC CCC-3′	Co-reduction	EF = 1.1 QY = 4.5%	Sulfide ions detection by quenching of luminescence The DNA-AuAgNCs provided higher sensitivity for S^2−^ than the DNA-AgNCs. Relative to DNA-AgNCs, DNA-AuAgNCs are much more stable in high ionic strength media DNA-AuAgNCs have a longer emission wavelength (630 vs. 530 nm)	[59]
Au-Ag	C_4_-ATAT-C_4_	Co-reduction	EF = N/A QY = 4.5%	Ratiometric detection of specific nucleic acid target. The probe exhibited high selectivity towards gastric cancer target	[74]
Au-Ag	C_12, 16, 20, 24_	Co-reduction	EF = N/A QY = N/A	Hg^2+^ detection by fluorescence quenching. Tunable emission	[75]
Cu-Ag	DNA: 5′CCCTTAATCCCC-3′	Cu^2+^ doping	EF = 4.5 relative to DNA–AgNCs QY = 51.2% Proposed mechanism: increasing k_r_, changing the nature of the HOMO-LUMO transition [this review]	SSB detection by fluorescence quenching	[55]
Cu-Ag	C-rich DNA	Co-reduction	EF = N/A QY = N/A	Hg^2+^ detection by fluorescence quenching; better selectivity and lower detection limit than that of the Ag NCs	[76]
Cu-Au	GSH	Galvanic replacement	~20× luminescence intensity vs. Cu NCs QY = N/A	Temperature sensor (20–70 °C) Detection of Cr^3+^ by quenching	[91]
Au-Ag	GSH	Co-reduction	EF = N/A QY = N/A	Detection of Hg^2+^ and Cu^2+^ by quenching. High photostability	[90]
Au-Ag	Glutathione S-transferase	Co-reduction	EF = 1.2 compared to GST-AuNCs QY = 13.5%	Detection and bioimaging of oxytetracycline	[96]

Theoretical studies of bimetallic and trimetallic NCs on biomolecules are very rare. It was shown that AuAg clusters demonstrated a strong affinity for cytosine nucleobases, primarily due to the basicity of the ring nitrogen atoms, which serve as the most favorable binding sites. While cytosine exhibited the strongest binding with pure gold clusters, within nanoalloys, it preferentially formed bonds with Ag atoms rather than gold due to the lower electronegativity of Ag. The Ag_4_Au_4_ cluster incorporated into a C-rich 9-nt hairpin loop revealed that these clusters can maintain overall stability even when interacting with massive DNA fragments [101].

Moreover, machine learning algorithms can be applied to the study of BNCs. For example, a nanosensor based on the GSH-AuAg clusters was developed for methotrexate detection. In doing so, deep machine learning with the use of NNets was applied for the analysis of data [102]. There is also a bright example of how BSA-protected AuAg clusters were developed as probes for the fluorescent sensing of tetracycline drug molecule. The authors used bagging, random forest, support vector machines, decision tree, as well as the artificial NNets methods not to study the clusters themselves but to analyze their images. As a whole, bagged artificial NNet and bagged decision tree models showed the best performance in assisting the detection of tetracycline [103].

In summary, Table 2 reveals a substantially less systematic picture for biopolymer-protected polymetallic NCs. In contrast to structurally resolved thiolate-protected clusters, most biomolecular systems exhibit modest enhancement factors, typically only a few-fold, and in most cases direct comparisons between polymetallic NCs and their monometallic counterparts are not reported. Moreover, quantitative comparisons are often complicated by differences in synthesis conditions, metal-to-ligand ratios, cluster composition, and sample heterogeneity. Importantly, the mechanisms underlying the reported QY enhancements are rarely investigated in detail. The few systems for which quantum yields and excited-state lifetimes can be compared provide particularly valuable mechanistic clues. For example, an increase in QY accompanied by a longer lifetime is consistent with suppression of nonradiative relaxation [69], whereas a substantial increase in QY accompanied by a shorter lifetime can indicate an important contribution from changes in the radiative rate [54,55,69]. In these cases, the corresponding interpretations are presented in this review.

Biopolymer-protected NCs have been widely explored as luminescent probes for the detection of various analytes, including metal ions, sulfide ions, cysteine, biothiols, folic acid, DNA, and proteins. Fluorescent thermometers and pH sensors have also been developed using these NCs. Higher QYs can improve the signal-to-background ratio and, consequently, the sensitivity of luminescence-based sensing. In some cases, polymetallic NC probes have exhibited higher selectivity or sensitivity toward specific analytes than their monometallic counterparts [75,76,81]. Enhanced chemical stability has also been reported for some polymetallic systems [59,74], which may further benefit their practical use.

Thus, Table 2 highlights both the considerable potential of biopolymer-protected polymetallic NCs for sensing and related applications and the current limitations in establishing the molecular mechanisms responsible for their enhanced luminescence.

## 4. Challenges and Future Directions

Despite substantial progress in developing bright polymetallic NCs, several challenges continue to limit a comprehensive understanding and rational optimization of their luminescence. First, the atomic structures of many biopolymer-protected NCs remain unresolved, making it difficult to distinguish the direct effects of metal composition from changes in nuclearity, atomic arrangement, ligand coordination, or aggregation. Combining spectroscopy with advanced structural methods, molecular simulations, and QM/MM approaches could help resolve these coupled effects. For example, detailed structural models of DNA–Ag [78,104] and protein-Ag [105,106] chromophore centers have been proposed using QM/MM-MD simulations of fluorescence excitation and excitation anisotropy spectra, together with comparisons of the calculated spectra with the corresponding experimental data. Such approaches demonstrate how the combination of molecular simulations and spectroscopic data can provide structural information for biopolymer-protected metal clusters even when direct atomic-resolution characterization is not available. Extending such approaches to polymetallic systems could be particularly valuable for determining whether metal incorporation primarily modifies the emissive electronic states, suppresses nonradiative relaxation, or alters intersystem crossing.

Second, systematic excited-state studies combining steady-state and time-resolved spectroscopy with theoretical calculations remain relatively scarce. Such investigations are needed to determine whether enhanced QY results primarily from suppression of nonradiative relaxation, enhancement of the effective radiative rate, modified intersystem crossing, or a combination of these processes. Carefully matched mono- and polymetallic reference systems will be particularly important for establishing causal structure–composition–photophysics relationships.

Another important challenge concerns the photostability of polymetallic NCs. While numerous studies report enhanced luminescence quantum yields upon metal alloying, considerably less attention has been devoted to understanding how polymetallic composition influences resistance to photodegradation. Photostability is a critical parameter for practical applications, including long-term bioimaging, continuous sensing, optical tracking, and light-emitting devices, where prolonged irradiation can induce irreversible structural and electronic changes that reduce luminescence intensity. Emerging evidence suggests that alloying may substantially improve photostability [69,107]. However, systematic studies correlating composition, structure, excited-state dynamics, and photostability remain scarce. Future work should therefore combine controlled photobleaching experiments with time-resolved spectroscopy and theoretical calculations to identify the key factors governing photodegradation pathways. Establishing quantitative structure–photostability relationships would not only improve the fundamental understanding of polymetallic NC photophysics but also provide guidelines for the rational design of bright and photostable luminescent nanoclusters.

Another challenge is the inconsistent reporting of optical performance. QY, emission intensity, extinction coefficients, and molecular brightness are not interchangeable parameters, and an increase in QY does not necessarily correspond to a proportional increase in brightness. Future studies should therefore report absorption or extinction coefficients together with QY whenever possible and use standardized experimental conditions for comparisons between mono- and polymetallic NCs. This would help distinguish intrinsic photophysical enhancement from differences in concentration, absorption, sample preparation, or cluster populations.

Finally, practical applications require more than high brightness. Photostability, chemical stability, reproducibility, resistance to biological media, and long-term performance should be evaluated under application-relevant conditions. Greater integration of atomically resolved characterization, excited-state spectroscopy, computational modeling, and data-driven approaches may ultimately enable predictive design of polymetallic NCs with optimized luminescence, stability, and biocompatibility.

## 5. Conclusions

This review examined how polymetallic composition influences the luminescence of metal nanoclusters, with particular emphasis on thiolate- and biopolymer-protected systems. A large number of polymetallic NCs with higher luminescence QYs than their monometallic counterparts have been reported, demonstrating that changes in metal composition can provide an effective route for increasing QY. In this review, we aim to identify experimentally supported microscopic mechanisms of luminescence enhancement, rather than empirical composition–luminescence correlations.

Excited-state dynamics are key to understanding the origin of luminescence enhancement. Comparison of QYs with excited-state lifetimes provides a useful first indication of the dominant photophysical contribution: increases in QY accompanied by longer lifetimes are consistent with suppression of nonradiative relaxation, whereas substantial QY increases accompanied by shorter lifetimes can indicate an important contribution from changes in radiative dynamics and/or the electronic character of the emissive state. Three main factors can be distinguished: suppression of nonradiative relaxation through rigidification of the cluster framework and/or ligand environment; modification of the electronic structure and emissive transition, which can alter the effective radiative rate; and enhanced intersystem crossing in systems where changes in spin–orbit coupling facilitate population of emissive triplet states. These contributions are frequently coupled rather than independent. For example, the same metal incorporation may simultaneously rigidify the structure, modify the frontier orbitals, and alter the rates of radiative, nonradiative, and intersystem-crossing processes. The Au_12_Ag_13_ system provides a particularly clear example in which changes in electronic structure and structural relaxation occur together [10,44], while Cu6Au16 demonstrates that enhanced ISC can coexist with strongly suppressed nonradiative decay [27].

The most convincing mechanistic assignments are obtained when structural characterization, absorption and steady-state spectroscopy, time-resolved measurements, and theoretical calculations are combined using closely matched monometallic and polymetallic reference systems. This requirement is particularly important because incorporation of a second metal can simultaneously change nuclearity, atomic arrangement, ligand coordination, charge state, and aggregation, making it difficult to isolate a direct metal-composition effect.

The analysis also reveals a pronounced difference between structurally characterized thiolate-protected clusters and biopolymer-protected NCs. For the former, atomic-level structures and increasingly sophisticated excited-state calculations provide a basis for relating specific metal sites and structural motifs to photophysical behavior. In contrast, the atomic structures of most biopolymer-protected polymetallic NCs remain unresolved, and comparative measurements of excited-state dynamics are scarce. As a result, the reported QY enhancements in these systems are generally more difficult to interpret mechanistically, even though they show considerable promise for sensing, bioimaging, and related applications. The available evidence indicates that higher QY, enhanced selectivity or sensitivity, and improved chemical stability can provide practical advantages, but these properties should not be assumed to arise from a common mechanism.

Looking forward, the field should move from empirical identification of highly emissive polymetallic NCs toward predictive structure–composition–photophysics relationships. This will require carefully matched mono- and polymetallic reference systems, atomically resolved structural characterization where possible, quantitative measurements of absorption and QY, time-resolved and ultrafast spectroscopy, and quantum-chemical or QM/MM modeling. For biopolymer-protected NCs, the integration of molecular simulations with spectroscopic observables offers a particularly promising approach for establishing structural models when direct atomic-resolution measurements are challenging. Such integrated studies should also examine photostability and performance under realistic sensing and biological conditions. Ultimately, combining control over metal composition, atomic structure, ligand organization, and excited-state dynamics should enable the rational design of polymetallic NCs that are not only brighter, but also more photostable, chemically robust, and biocompatible, thereby translating molecular-level photophysical insights into improved sensing and bioimaging technologies.

## Figures and Tables

**Table 1:** Thiolate-protected BNCs exhibiting enhanced luminescence.

Metal Core	Ligand	Method of Synthesis	QY Enhancement Factor (EF), QY, Optical Features	Mechanism of QY Enhancement	Reference
Au_15_@Ag, Au_18_@Ag, and Au_25_@Ag	(GSH)_13_, (GSH)_14_, and (GSH)_18_, respectively	Doping with Ag(I) ions	EF = 84 for Au15@Ag QY = 2.1% EF = 18 for Au18@Ag QY = 6.8% EF = 27 for Au25@Ag QY = 3.2%	Rigidifying the framework (AIE effect)	[40]
Ag_x_ Au_25−x_ (x = 1–13)	(PPh_3_)_10_ (SC_2_H_4_ OH)_5_Cl_2_, where SC_2_H_4_ OH is a hydroxyethylthiol	Co-reduction	EF = 200 for Ag_13_Au_12_ QY = 40%	Changing the nature of the HOMO-LUMO transition [10,44]; increasing k_r_ and decreasing k_nr_ [44]; rigidifying the NC [44,45]	[10]
Au_25−n_Ag_n_ (*n* = 0–11)	(SC_12_H_25_)_18_	Co-reduction	EF ~ 1 QY = N/A Modulation of emission wavelength	Incorporation of Ag continuously modulates the electronic structure of the Au_25_ NC	[46]
Ag_29−x_Au_x_ , x = 1–7	(BDT)_12_ (TPP)_4_, where TPP is triphenylphosphine and BDT is 1,3-benzenedithiolate	Co-reduction	EF = 26 QY = 24% for 40% Au-doped samples	A strong perturbation to the electronic structure [6]; increasing k_r_ and decreasing k_nr_ [this review]; relativistic effects [47]	[6]
M_29_, where M = Ag/Cu/Au/Pt/Pd	(S-Adm)_18_(PPh_3_)_4_, where S-Adm is the adamantane mercaptan	Metal exchange	EF > 2 for Au_5_Ag_24_ QY = 11.6%	A change in metal composition probably changes the electronic structure [this review]	[19]
Pt_1_Ag_28_	(BDT)_12_(TPP)_4_	Ligand exchange	EF = 2.3 QY = N/A	Electronic structure modification [48]; suppression of nonradiative relaxation [this review]	[48]
Pt_1_Ag_24_ and Pt_1_Ag_28_	(SPhMe_2_)_18_ and (S-Adm)_18_(PPh_3_)_4_	Thiol etching	EF > 5 for Pt_1_Ag_28_ vs. Ag_29_ EF = 50 for Pt_1_Ag_28_ vs. Pt_1_Ag_24_ QY = 4.9%	Suppressing nonradiative pathways	[49]
Cu_6_Au_16_	(tBuPhC≡C)_18_, where tBu represents tert-butyl and Ph stands for phenyl	Ligand exchange	EF ~ 10 QY = 95%	Suppressing nonradiative pathways and enhancing ISC	[27]
Cu_1_Au_38_	(2-PET)_24_, where PET is a 2-phenylethanethiol	Metal exchange	EF ~ 2 QY = N/A	N/A	[12]
M@Cu_12_, where M = Ag or Au	(Dithiolate)_6_ (C≡CPh)_4_, where dithiolate = dithiocarbamate, dithiophosphate, or dithiophosphinate	Galvanic replacement	EF = 100 for AuCu_12_ relative to AgCu_12_ QY = 55% ≫Cu@Cu_12_	Enhancing spin–orbit coupling	[50]
Au-Cu_14_	[(SR)_12_(BCPP)_6_]^+^ SR-4-tert-butyl-benzenethiolate, BCPP-bis(2- cyanoethyl)-phenylphosphine	Co-reduction	EF = 1.7 relative to ClCuNCs QY = 71.3%	Enhancing spin–orbit coupling [51] Suppressing nonradiative pathways [this review]	[51]
Au2Cu6	(PPh2Py)2- (SC10H15)	Selective reduction of Au complexes	EF = N/A (Monometallic CuNCs are non-luminescent) QY = 11.7%	Au0-induced aggregation of the CuSR complexes	[43]

## Data Availability

The original contributions of this study are included in this manuscript, and further inquiries can be directed to the corresponding author.

## Abbreviations

The following abbreviations are used in this manuscript:

BNCs	Bimetal nanoclusters
BSA	Bovine serum albumin
EF	Enhancement factor
HOMO	Highest occupied molecular orbital
GSH	Glutathione
LOD	Limit of detection
NCs	Nanoclusters
NIR	Near infra-red
MNCs	Monometallic nanoclusters
QY	Quantum yield
SG	Thiol
TD-DFT	Time-dependent density functional theory
